# Targeting Kinase Signaling in Glioblastoma: Structural Optimization, Blood–Brain Barrier Dynamics and Combinatorial Translational Strategies

**DOI:** 10.3390/ijms27156590

**Published:** 2026-07-24

**Authors:** Diana Juanes-Gusano, Beatriz Fernández-Roldán, Rafael Coveñas, Maruan Hijazi

**Affiliations:** 1 Group USAL-BMD (Bases Moleculares del Desarrollo), University of Salamanca, 37007 Salamanca, Spain; diguju@usal.es (D.J.-G.); fernandezr.bea@usal.es (B.F.-R.); 2 Institute of Neuroscience of Castilla and León (INCyL), iBRAINS-IN-CyL, 37007 Salamanca, Spain; 3 Institute of Biomedical Research of Salamanca (IBSAL), 37007 Salamanca, Spain

**Keywords:** glioblastoma, kinase inhibitors, blood–brain barrier, efflux transporters, structural optimization, prodrugs, combinatorial therapy, patient-derived xenografts

## Abstract

Small-molecule kinase inhibitors offer a compelling therapeutic strategy for glioblastoma, yet their clinical efficacy remains severely limited by blood–brain barrier penetration and active efflux transporter extrusion. This review evaluates current medicinal chemistry approaches and translational paradigms to overcome these drug delivery and biological constraints. A critical analysis of the literature reveals that direct structural optimization faces a multidimensional balancing act; next-generation design must prioritize macrocyclization, structural rigidification, and bioisosteric capping to lower polar surface area and evade P-glycoprotein and BCRP efflux. Furthermore, carrier-mediated prodrugs targeting the LAT1 transporter provide a viable rescue strategy for highly potent scaffolds. Reviewing recent clinical failures, such as paxalisib and osimertinib, underscores that single-node monotherapies fail due to compensatory pathway hyperactivation and clonal heterogeneity, whereas multi-targeted agents or rational dual-node combinations prevent rapid tumor adaptation. Additionally, combining kinase inhibitors with DNA damage repair inhibitors, immune checkpoint modulation, or MR-guided focused ultrasound could provide powerful synergistic networks. Finally, bridging the translational gap requires complementing conventional serum-cultured cell lines with patient-derived glioma stem cells and orthotopic xenografts to better recapitulate the cellular architecture of the disease. Ultimately, overcoming the therapeutic challenges in glioblastoma demands a fundamental pivot toward rigorous neuro-pharmacological design and multi-lineage network oncology.

## 1. Introduction

Gliomas account for nearly 80% of primary central nervous system (CNS) tumors. The 2021 World Health Organization classification (WHO CNS5) categorizes adult-type diffuse gliomas into three molecularly defined groups based on isocitrate dehydrogenase (IDH) mutation and 1p/19q codeletion status: astrocytoma, IDH-mutant; oligodendroglioma, IDH-mutant and 1p/19q-codeleted; and glioblastoma (GB), IDH-wildtype [1]. While IDH-mutant tumors typically progress from lower-grade lesions and carry a better prognosis, IDH-wildtype tumors develop via aggressive genetic pathways from early stages, presenting as de novo high-grade malignancies [2]. GB is the most common malignant brain tumor, representing 48.6% of cases [3], with an incidence of 3.26 per 100,000 people in developed countries [4]. It exhibits highly invasive behavior and rapid progression, resulting in a median survival of 15 months [5], a five-year survival rate of 6.9% [4], and severe neurological morbidity [6].

The standard multimodal treatment established by Stupp et al. combines surgical resection, radiotherapy (RT), and chemotherapy with temozolomide (TMZ) [7]. However, prognosis remains poor due to high recurrence rates, driving research into targeted therapies that inhibit specific cell cycle and proliferation proteins. Kinases have emerged as primary therapeutic targets because of their vital role in oncogenic signaling and their multi-tumor efficacy [8]. This review analyzes the potential of oral kinase inhibitors in GB, examining the challenges imposed by the tumor microenvironment (TME) and the blood–brain barrier (BBB), altered molecular signaling cascades, and pharmacological strategies to overcome current therapeutic limitations.

## 2. General Features of Glioblastoma

Under the WHO CNS5 framework, GB is defined as an IDH-wildtype entity arising de novo, whereas high-grade diffuse gliomas progressing from lower-grade precursors are classified as astrocytomas, IDH-mutant, grade 4. The cellular origin of GB involves two primary models: the glioma stem cell (GSC) theory and the astrocyte dedifferentiation hypothesis [9]. The GSC theory suggests that quiescent stem cells drive tumor plasticity and heterogeneity; these cells frequently derive from neural stem cells (NSCs) [10] and share mutations (TERT, PTEN, EGFR, TP53) and markers (Sox10, nestin, vimentin, GFAP, Olig1/2). Conversely, the dedifferentiation hypothesis posits that differentiated astrocytes re-acquire stem-like properties following oncogenic activation [9]. Both models explain the presence of GSCs, which mediate tumor growth, immunosuppression, and therapy resistance.

**Figure 1:**
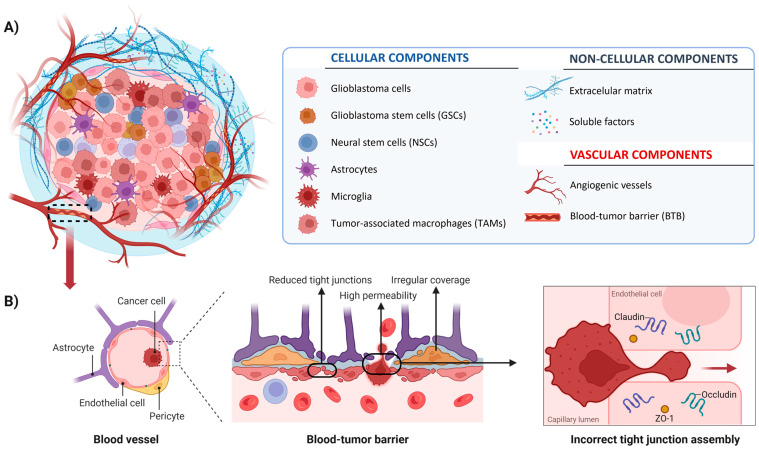
Glioblastoma microenvironment and structural characteristics of the blood–tumor barrier (BTB). GB exhibits significant intratumoral heterogeneity, arising from the coexistence of distinct tumor cell populations and diverse microenvironmental compartments. (**A**) Dynamic interactions among tumor cells, stem-like cells, vascular components, immune infiltrates, stromal elements, and extracellular matrix components generate spatially and functionally distinct niches that promote tumor adaptation, invasion, immune evasion, and therapeutic resistance. (**B**) In GB, BBB disruption leads to the formation of the BTB, which is characterized by heterogeneous permeability, reduced tight junction integrity, abnormal pericyte coverage, and angiogenic remodeling. Tight junction proteins such as claudins and occludin are anchored to the cytoskeleton through zonula occludens proteins (ZO) to restrict paracellular transport. Therefore, BTB structural abnormalities contribute to heterogeneous drug distribution within the tumor, limiting therapeutic efficacy and driving treatment failure. Created in BioRender. Hijazi Vega, M. (2026) https://BioRender.com/d0m8d4d (accessed on 16 July 2026).

Histopathologically, GB is characterized by high mitotic activity, diffuse tissue invasion, microvascular hyperplasia (angiogenesis), and necrosis [2,11]. Hypoxia within the tumor core induces stress-adaptation mechanisms that enhance tumor aggressiveness, while tumor cells modulate the TME to foster local immunosuppression [12]. The TME comprises a specialized parenchymal network of neurons, astrocytes, and microglia interacting with neoplastic cells [13] (Figure 1). Spatially, the tumor core drives angiogenic signaling via GSC-derived VEGF secretion within vascular niches [14], while tumor-associated macrophages (TAMs) adopt highly plastic, immunosuppressive activation states rather than a strict M1/M2 polarization [15]. Activated astrocytes further support tumor migration and immunosuppression by releasing cytokines like TGF-β, IL-10, and G-CSF.

The BBB protects the CNS via the neurovascular unit, which integrates cerebral endothelial cells, pericytes, astrocytes, and a basement membrane [16,17]. Endothelial cells utilize tight junctions (claudins, occludin, JAMs connected to the cytoskeleton via ZO-1/ZO-2) to prevent the passive passage of hydrophilic molecules [16,17]. Astrocytes maintain barrier integrity by releasing regulatory factors (GDNF, TGF-β, Sonic Hedgehog), while pericytes regulate vascular stability [16]. In GB, the BBB is structurally altered into a heterogeneous blood–tumor barrier (BTB) (Figure 1); permeability increases in the angiogenic, hypoxic tumor core due to VEGF-driven neovascularization, whereas the barrier remains largely intact at the infiltrative peripheral margins [18,19,20]. Furthermore, active functional efflux is driven by ATP-binding cassette (ABC) transporters, such as P-glycoprotein (P-gp), breast cancer resistance protein (BCRP), and multidrug resistance-associated proteins (MRPs), which return lipophilic compounds to the bloodstream [21]. Because most kinase inhibitors are substrates for P-gp and BCRP, bypassing these active efflux mechanisms is a critical constraint for drug delivery [21].

## 3. Conventional Therapies

### 3.1. Current Management of Glioblastoma

Standard GB treatment combines maximal safe surgical resection, radiotherapy, concomitant/adjuvant TMZ, and Tumor Treating Fields [7]. Cytoreductive surgery prioritizes tumor mass reduction while preserving neurological integrity [22,23,24]. Although diffuse cell infiltration prevents complete surgical eradication [2], intraoperative adjuncts like intraoperative MRI (iMRI) and fluorescence-guided surgery (FGS) with 5-aminolevulinic acid (5-ALA) significantly maximize the extent of resection (EOR) [24]. Post-operative RT delivers 60 Gy in 2-Gy fractions over six weeks [6], inducing DNA strand breaks and reactive oxygen species (ROS) [25]. In response to sub-lethal radiation, cells activate survival mechanisms including the DNA damage response (DDR), autophagy, and the unfolded protein response [26], necessitating precise dosing to limit normal tissue toxicity [27].

Chemotherapy integration via the Stupp protocol established that adding oral TMZ to RT provides a 2.5-month survival benefit, extending median overall survival (OS) from 12.1 to 14.6 months [7]. Other agents, such as bevacizumab, prolong progression-free survival (PFS) but do not extend OS [28]. TMZ acts as an alkylating agent by inducing O6-methylguanine DNA lesions [4]. Its efficacy depends on MGMT promoter methylation status; epigenetic methylation silences the MGMT repair gene, preventing the removal of alkyl groups and rendering cells sensitive to chemotherapy [29]. Conversely, unmethylated MGMT promoters confer a resistant phenotype, resulting in minimal survival benefit from TMZ and making MGMT a key predictive biomarker [30].

### 3.2. Therapy Limitations and Recurrence

While clinical nomograms utilize variables like residual tumor volume, age, and MGMT status to estimate outcomes [31], median OS remains poor, and recurrence typically occurs within 6–9 months [32]. Relapse is driven by intratumoral epigenetic heterogeneity, GSC-mediated DNA repair, tissue acidosis, and reduced cell proliferation under hypoxic conditions [11,33]. RT-induced vascular damage further exacerbates hypoxia, activating adaptive radioresistance cascades that predict poor clinical outcomes [8,34]. Additionally, while TMZ achieves high bioavailability and crosses the BBB (≥30% in CSF) [30], approximately 98% of small-molecule candidates during industrial development pipelines fail to reach therapeutic concentrations in the brain parenchyma [20,35]. Because non-specific genotoxic therapies select for highly aggressive clones, the paradigm may shift toward targeted kinase inhibitors (KIs) designed to cross the BBB and selectively disrupt survival networks in both differentiated cells and quiescent GSCs.

## 4. Kinases and Signaling Pathways

### 4.1. Protein Kinases in Tumor Signaling

Kinases regulate cellular homeostasis by catalyzing the transfer of a phosphate group from ATP to specific serine, threonine, or tyrosine residues on target proteins [33,36]. This reversible molecular switch controls cell division, proliferation, metabolism, and apoptosis [36]. Dysregulation of kinase activity disrupts conformational signaling and intercellular communication, driving uncontrolled cell division and tumorigenesis [37].

### 4.2. Key Signaling Pathways and Molecular Classification of Glioblastoma

Kinases are classified into receptor tyrosine kinases (RTKs: EGFR, PDGFR, FGFR, MET, VEGFR) and non-receptor kinases (cytoplasmic/nuclear mediators like PI3K, AKT, mTOR, RAS, RAF, MEK, ERK) [37]. In GB, persistent kinase signaling converges on identical downstream effectors, creating functional redundancies that allow tumors to maintain growth when a single pathway is blocked [33,37].

The Cancer Genome Atlas (TCGA) project identified distinct molecular subtypes of GB based on genomic alterations: classical (EGFR amplification/mutation, CDKN2A loss, hyperactivated Notch/Hedgehog); mesenchymal (NF1 and PTEN mutations, MET overexpression, pro-inflammatory TME); and proneural (PDGFRA alterations, TP53, and IDH1 mutations) [38,39,40]. Multi-omic refinements demonstrated that the previously described neural subtype is an artifact caused by normal tissue contamination during bulk sequencing [39,40]. Modern single-cell RNA sequencing (scRNA-seq) has replaced this static paradigm, establishing that GB displays profound intratumoral heterogeneity where four distinct, plastic cellular states: astrocytic (AC-like), oligodendrocyte progenitor (OPC-like), neural progenitor (NPC-like), and mesenchymal (MES-like), coexist dynamically within the same tumor and shift phenotypes in response to therapeutic pressure [41].

### 4.3. The Role of Signaling Pathways in Glioblastoma Recurrence

The genomic complexity of GB involves the simultaneous activation of multiple interconnected cascades rather than a single dominant driver [8,33]. EGFR amplification and the constitutively active EGFRvIII mutant trigger ligand-independent signaling through the PI3K/AKT/mTOR and RAS/RAF/MEK/ERK pathways [33,37]. In the PI3K axis, AKT activation inhibits pro-apoptotic proteins and stimulates mTOR-mediated metabolism; this cascade is frequently intensified by the loss or inactivation of the tumor suppressor PTEN, leading to sustained survival and treatment resistance [8,33,37]. Parallelly, the MAPK pathway is hyperactivated by RTK upstream signaling or mutations in the negative regulator NF1, where downstream ERK activation drives gene expression linked to cell cycle progression, invasion, and relapse [37].

This signaling network is further destabilized by TP53 mutations, which impair the p53-mediated DNA damage response, accelerating the accumulation of mutations and increasing intratumoral heterogeneity [37]. Co-existing alterations in the RB pathway and alternative RTKs (PDGFR, MET, FGFR) enable rapid adaptive bypass; for instance, blocking EGFR frequently induces the compensatory upregulation of MET or PDGFR, rendering single-target therapies ineffective [37,42]. Additionally, quiescent GSCs exploit conserved pathways (Notch, Wnt, Hedgehog) to maintain self-renewal and drive post-treatment recurrence [42], while microenvironmental stressors like chronic hypoxia activate the NF-κB and JAK/STAT pathways to enhance cell survival and blunt genotoxic therapies [33].

## 5. Targeted Therapies: Kinase Inhibitors (KIs)

### 5.1. Types of Kinase Inhibitors

Small-molecule protein kinase inhibitors (smKIs) represent a major category of targeted therapeutic agents that are designed to disrupt aberrant kinase signaling, a characteristic that is present in the pathobiology of GB. These agents are typically low-molecular-weight organic compounds and lipophilic, penetrating cells and directly targeting the catalytic activity of intracellular kinases. Given that kinase function depends on the coordinated binding of ATP and protein substrates to enable phosphate transfer, the fundamental principle underlying smKI design is the disruption of ATP binding, substrate interaction, or kinase conformational dynamics, consequently impeding downstream oncogenic signaling. In GB, where receptor tyrosine kinases and intracellular signaling cascades are frequently dysregulated or constitutively activated, such inhibition aims to suppress tumor cell proliferation, survival, and invasion [43,44,45,46,47].

**Figure 2:**
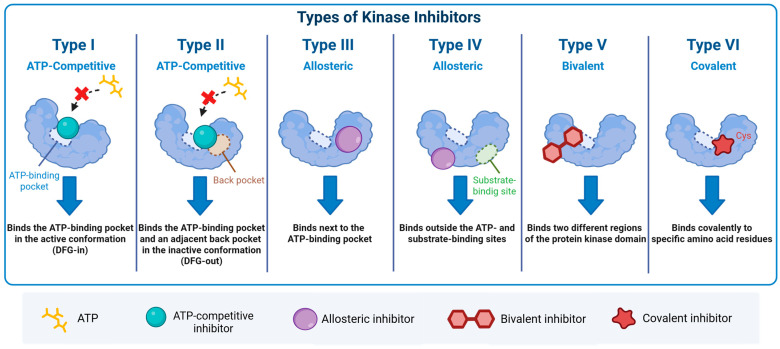
Classification of small-molecule protein kinase inhibitors according to their binding mode. ATP-competitive inhibitors include type I and I ½ inhibitors, which bind to the ATP-binding pocket in the active DFG-in kinase and inactive DFG-Asp in conformation, respectively. Then, type II inhibitors, which recognize the inactive DFG-out kinase conformation by occupying the ATP-binding pocket and an adjacent hydrophobic back pocket. Allosteric inhibitors comprise type III inhibitors, which bind next to the ATP-binding pocket, and type IV inhibitors, which do not bind to the ATP- or peptide substrate-binding sites. Type V inhibitors bind to two different regions of the protein kinase domain and are therefore bivalent inhibitors, whereas type VI inhibitors bind covalently to specific amino acid residues, typically cysteine or lysine. Created in BioRender. Hijazi Vega, M. (2026) https://BioRender.com/ntcukpb (accessed on 16 July 2026).

Mechanistically, the classification of smKIs is determined according to their mode of binding to the kinase domain and their interaction with either the ATP-binding site or alternative regulatory regions (Figure 2). The most established strategy of kinase inhibition is ATP-competitive inhibition, which exploits the conserved ATP-binding cleft located between the N- and C-terminal lobes of the kinase catalytic domain [48]. Type I kinase inhibitors bind reversibly to the active (DFG in) conformation of kinases, directly occupying the ATP-binding pocket by interacting with residues in the hinge region and mimicking the purine ring of ATP [49,50,51]. Type I½ inhibitors represent a distinct intermediate class that binds to an inactive conformation where the DFG motif remains in (DFG in), but the αC-helix is displaced. In contrast, type II inhibitors recognize and stabilize the classic inactive conformation (DFG out), extending into adjacent hydrophobic pockets exposed by the flipped motif, a mechanism that sterically hinders ATP binding and often increases selectivity due to the structural variability of inactive states [52,53].

Beyond ATP-competitive compounds, allosteric inhibitors bind to sites distinct from the ATP pocket, modulating activity via conformational changes. These are categorized into type III inhibitors, which interact with allosteric regions in close proximity to the ATP-binding site [54], and type IV inhibitors, which bind to distant allosteric sites completely outside the catalytic cleft [55]. Furthermore, type V inhibitors comprise bivalent or bisubstrate compounds that engage multiple regions of the kinase domain simultaneously, spanning across both the ATP site and an adjacent regulatory pocket [54]. Finally, type VI inhibitors form covalent interactions, either reversible or irreversible, with specific nucleophilic residues, typically targeting the thiol groups of cysteine residues within or near the active site to achieve sustained therapeutic blockade [55,56,57].

This classification reflects the intrinsic structural plasticity of kinase domains, including conformational changes in key motifs such as DFG and the αC-helix, and provides a framework for the rational design of inhibitors. In GB, where kinase-driven signaling pathways are frequently dysregulated and highly adaptive, this mechanistic diversity is particularly relevant to overcome therapeutic resistance and improve target selectivity within the CNS.

It is important to note that not all kinase-targeting therapies are small molecules. In addition to small molecule kinase inhibitors, monoclonal antibodies (mAbs) represent a distinct class of inhibitors that act through a fundamentally different mechanism. In contrast to small molecules, mAbs represent large, protein-based therapeutic agents that do not penetrate the cell membrane; rather, they bind to the extracellular domain of receptor tyrosine kinases (RTKs). This interaction has been shown to block ligand binding, prevent receptor dimerization and activation, and promote receptor internalization and degradation, thereby inhibiting downstream signaling pathways. This complementary approach is particularly relevant for targeting dysregulated surface receptors in cancer, including GB, and highlights the broader therapeutic landscape beyond intracellular ATP-competitive inhibition.

### 5.2. Critical Physicochemical Properties for Oral Bioavailability of KIs and Central Nervous System Penetration

The design of KIs that are capable of effectively targeting GB necessitates meticulous optimization of physicochemical properties that facilitate both oral bioavailability and penetration across the BBB. In contrast to the numerous first-generation oncology drugs that were not originally developed with CNS exposure in mind, next-generation inhibitors must achieve a delicate balance between permeability, solubility, metabolic stability and efflux liability in order to attain therapeutically relevant unbound drug concentrations in the brain. Among the most critical determinant factors are molecular weight, lipophilicity, polarity, and ionization state, all of which influence passive transcellular diffusion and susceptibility to active efflux transporters, such as P-gp [58,59].

In this context, molecular weight (MW) represents a primary determining factor of membrane permeability. Most clinically relevant kinase inhibitors typically fall within the range of approximately 380–500 Da, which is considered close to the upper limit compatible with efficient passive diffusion. Excessive increases in MW are generally associated with reduced intestinal absorption, reduced BBB permeability and enhanced recognition by efflux transporters, ultimately limiting CNS exposure. Consequently, medicinal chemistry optimization strategies frequently involve the elimination of redundant substituents or the substitution of bulky aromatic moieties with more compact heterocyclic scaffolds in order to preserve permeability, whilst preserving target affinity [59,60,61].

Closely associated with molecular size, lipophilicity constitutes another critical parameter governing both oral absorption and BBB penetration; while logP describes the partition coefficient of the neutral species between octanol and water, logD more accurately reflects in vivo behavior by incorporating ionization at physiological pH. In general, moderate lipophilicity (typically logP ≈ 2–4) is considered optimal for kinase inhibitors, as it facilitates passive membrane diffusion without excessively compromising aqueous solubility. Indeed, compounds displaying excessively high lipophilicity often exhibit poor aqueous solubility, increased metabolic clearance, and higher affinity for efflux transporters. Conversely, highly polar molecules (low logP) tend to display limited membrane permeability. Accordingly, fine-tuning lipophilicity through subtle structural modifications, including the incorporation or removal of aromatic substituents, halogens or polar heterocycles, has become a central strategy in modern CNS-oriented drug design [59,62,63,64].

Furthermore, molecular polarity, which is typically quantified by topological polar surface area (TPSA) together with the number of hydrogen bond donors and acceptors, plays a critical role in BBB permeability of the KIs. Lower polarity is generally associated with enhanced passive diffusion, as evidenced by TPSA values below approximately 90 Å^2^ and a limited number of hydrogen bonding interactions correlating with improved CNS penetration. Conversely, an excess of polar functional groups, particularly hydroxyl and amine moieties (OH, NH), increases solvation and reduces membrane permeability. Accordingly, medicinal chemistry strategies frequently aim for a reduction in hydrogen bonding capacity through functional group modification or the incorporation of less polar bioisosteres, thereby enhancing CNS exposure while preserving biological activity [61,62,63].

Importantly, ionization state and pKa are also intimately linked to membrane permeability and CNS distribution. Since neutral molecular species diffuse more efficiently across lipid bilayers than charged forms, optimization of pKa to minimize ionization at physiological pH represents a key objective in CNS drug discovery. Charged compounds not only exhibit reduced passive diffusion but are also more susceptible to active efflux mediated by transporters such as P-gp. To address these limitations, medicinal chemists commonly modulate the basicity of amine-containing groups, introduce electron-withdrawing substituents such as chlorine or trifluoromethyl groups (Cl or CF3), or incorporate heterocyclic scaffolds that stabilize the neutral form of the molecule. Importantly, ionization state is tightly interconnected with both lipophilicity and polarity, underscoring the necessity for simultaneous optimization of these parameters to achieve an appropriate balance between solubility, permeability and CNS exposure [59,62,63,65].

Finally, molecular flexibility, typically estimated by the number of rotatable bonds, constitutes an additional determinant of oral bioavailability and membrane permeability. Molecules with reduced conformational flexibility generally exhibit improved passive diffusion across biological membranes and more favorable pharmacokinetic profiles, supporting enhanced CNS exposure [64,66]. Consequently, restricting molecular flexibility through conformational constraint strategies has emerged as an alternative approach to optimize the brain penetration profile of kinase inhibitors.

**Figure 3:**
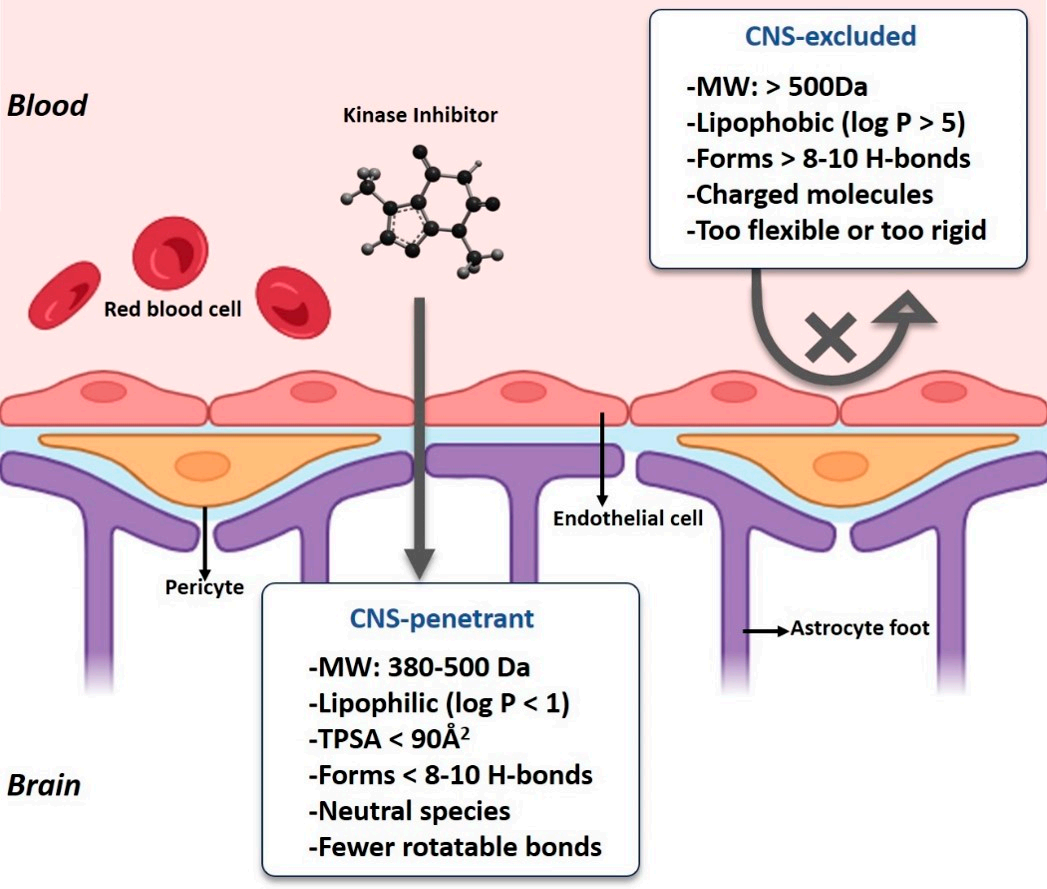
The blood–brain barrier (BBB). The BBB is a highly selective neurovascular interface formed by endothelial cells joined by tight junctions, supported by pericytes and astrocytic end-feet, which collectively regulate molecular trafficking into the central nervous system. Effective CNS-active kinase inhibitors require physicochemical properties that favor passive diffusion and limit efflux, including relatively low molecular weight, moderate lipophilicity, reduced polarity (low TPSA and hydrogen bonding capacity), and minimal recognition by transporters such as P-glycoprotein and BCRP. Only compounds meeting these criteria can achieve sufficient brain penetration to exert therapeutic effects in glioblastoma. Created in BioRender. Hijazi Vega, M. (2026) https://BioRender.com/e87cgjj (accessed on 16 July 2026).

Collectively, the pharmacokinetic and distribution profile of kinase inhibitors is defined by the collective interplay between molecular size, lipophilicity, polarity, and ionization. In the context of GB, where effective drug delivery to the brain remains a significant therapeutic challenge, rational optimization of these physicochemical properties is recommended to enhance BBB penetration and improve clinical efficacy. Figure 3 summarizes the key physicochemical characteristics that kinase inhibitors should possess in order to facilitate effective BBB permeation.

### 5.3. Chemical Design of Oral Kinase Inhibitors

The therapeutic efficacy of orally administered kinase inhibitors in GB is critically determined by their chemical design, which must simultaneously ensure high affinity for the kinase catalytic domain and favorable pharmacokinetic properties compatible with CNS exposure. In contrast to inhibitors developed for peripheral tumors, compounds targeting brain malignancies must be specifically engineered to balance selectivity, oral bioavailability and the ability to penetrate the BBB. This imposes a stringent set of structural and physicochemical constraints, thus rendering rational drug design a central determinant of clinical success.

At the molecular level, the majority of kinase inhibitors are designed to occupy the ATP-binding pocket within the catalytic domain, thereby preventing phosphate transfer and downstream signaling. This is typically achieved through the use of heterocyclic scaffolds that mimic the adenine moiety of ATP and establish key hydrogen-bond interactions with the hinge region of the kinase. From this core, chemical substituents are strategically introduced to extend into adjacent subpockets, modulating binding affinity, selectivity, and pharmacokinetic behavior. Structurally, these compounds can also be understood in relation to the conformational state of the kinase they target. Inhibitors that bind the active conformation generally adopt compact, planar architectures optimized for direct interaction within the ATP site. In contrast, those targeting inactive conformations often incorporate more extended or flexible substituents to access additional hydrophobic regions and stabilize alternative kinase states [67,68].

**Figure 4:**
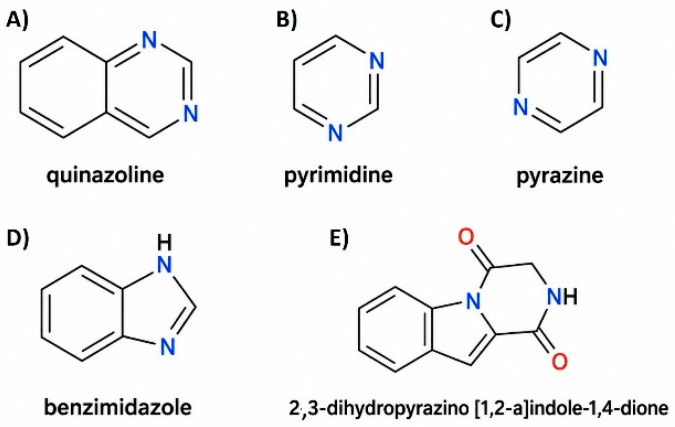
Chemical design of kinase inhibitors. Representative heterocyclic scaffolds commonly used in kinase inhibitor development: (**A**) quinazolines, (**B**) pyrimidines, (**C**) pyrazines, (**D**) benzimidazoles, and (**E**) 2,3-dihydropyrazino [1,2-a]indole-1,4-dione as an example of hybrid and fused heterocyclic systems. These cores act as ATP-mimetic frameworks that enable hinge binding and can be functionally decorated to modulate affinity, selectivity, and pharmacokinetic properties relevant for CNS-targeted therapies.

Quinazolines represent a particularly prominent molecule among the most widely used scaffolds, and they are utilized extensively in the field, particularly in the context of receptor tyrosine kinase inhibitors. The fused aromatic structure, which contains two nitrogen atoms, enables the formation of key hydrogen bonds with the hinge region of the kinase domain that recapitulate ATP binding (Figure 4A). Furthermore, the aromaticity of the quinazoline core offers multiple positions for the incorporation of electronically active substitutions (halogens, alkyl groups, amines, or additional rings), enabling the introduction of substituents that modulate potency, selectivity, and physicochemical properties such as lipophilicity and pKa. This tunability has facilitated the development of analogues with enhanced oral bioavailability and optimized kinase selectivity profiles [62,67,69,70].

Similarly, pyrimidine- (Figure 4B) and pyrazine-based (Figure 4C) drugs, including fused derivatives such as pyrazolopyrimidines or pyridopyrimidines, are widely employed as purine bioisosteres and serve as effective hinge-binding motifs. Their relatively small size and electronic versatility enable fine control over hydrogen bonding capacity and ionization state, while their substitution patterns allow the projection of functional groups into adjacent hydrophobic pockets. This structural adaptability facilitates the design of both selective and multi-target inhibitors, a feature particularly relevant in GB, where pathway redundancy often necessitates broader kinase inhibition [67,71,72,73,74].

Benzimidazoles constitute another important class of ATP biomimetic structures, offering favorable geometric complementarity with the kinase catalytic domain (Figure 4D). As functional bioisosteres of purines, they promote efficient binding to the hinge region, allowing modulation of polarity (by introducing OH/NH groups) and lipophilicity (by adding aromatic chains) through peripheral substitutions. This flexibility enables precise tuning of pharmacokinetic properties, including oral absorption and penetration into the CNS, crucial factors for therapeutic efficacy in brain tumors [75,76,77].

Recent advances in medicinal chemistry have focused on the development of hybrid and fused heterocyclic systems, which integrate multiple pharmacophoric elements within a single molecular structure. These scaffolds combine rigid cores with strategically positioned functional groups, thereby enabling simultaneous interaction with multiple kinase domain subpockets and enhancing binding affinity to EGFR and BRAF active sites. In particular, fused systems incorporating indole-, pyrazine-, or pyrimidine-based motifs (as 2,3-dihydropyrazino [1,2-a]indole-1,4-dione, Figure 4E) provide a structurally constrained yet adaptable platform capable of optimizing both target interaction and pharmacological properties. These hybrid designs also facilitate the modulation of key parameters such as molecular weight, lipophilicity, and hydrogen-bonding capacity, thereby promoting oral bioavailability while maintaining the potential for CNS exposure. Taken together, these results demonstrate that this hybrid scaffold is a promising platform for the development of novel dual inhibitors with potential applications in cancer therapies [62,78,79].

### 5.4. Influence of Molecular Structure on Efflux Transporter Interaction (P-gp, BCRP)

The structure–activity relationship (SAR) of kinase inhibitors not only governs target affinity, but also critically determines their ability to achieve effective CNS exposure. In the context of GB, this balance is particularly challenging, as orally administered kinase inhibitors must overcome active efflux mediated by P-glycoprotein and breast cancer resistance protein, which are highly expressed at the BBB. Consequently, even minor structural modifications have the potential to enhance kinase inhibition while simultaneously increasing recognition by efflux transporters, ultimately limiting brain accumulation and therapeutic efficacy [59,62].

In this regard, heterocyclic scaffolds such as quinazolines, pyrimidines, pyrazines and benzimidazoles have become a prevalent component in kinase inhibitor design because of their ability to establish key hydrogen-bond interactions with the hinge region of the kinase domain. However, these same structural features (including planarity, aromaticity, heteroatom content, and hydrogen-bond acceptor capacity) are also frequently associated with increased substrate recognition by P-gp and BCRP. As a consequence, many ATP-competitive inhibitors display excellent biochemical potency yet poor CNS retention, illustrating a fundamental trade-off between binding efficiency and brain penetration.

Importantly, these physicochemical and structural attributes are intimately connected with transporter recognition at the BBB. In particular, aromaticity, lipophilicity and the presence of basic heteroatoms strongly influence substrate affinity toward P-gp and BCRP, leading to active extrusion from the CNS despite otherwise favorable pharmacological profiles. Accordingly, numerous kinase inhibitors exhibit suboptimal intracranial exposure even when their inhibitory potency against the molecular target is highly efficient [80].

Moreover, structure–activity relationship studies have demonstrated that even minimal modifications within aromatic regions can significantly alter efflux susceptibility. For example, in 4-anilinoquinazoline and quinoline-based inhibitors, alterations in the aniline substituent (including the introduction of hydroxylamine functionalities) have been shown to markedly reduce P-gp-mediated efflux without compromising kinase inhibitory activity [81]. These observations underscore how subtle modulation of hydrogen-bonding capacity, steric environment and electronic distribution can profoundly influence BBB permeability without necessarily compromising target affinity.

Beyond the heterocyclic core itself, sidechain functionalization also plays a decisive role in modulating both pharmacodynamic and pharmacokinetic properties. Indeed, peripheral substituents can significantly alter lipophilicity, polarity and molecular flexibility, thereby influencing transporter recognition. For instance, the introduction of halogen atoms (e.g., Cl, F) typically increases lipophilicity and enhances hydrophobic interactions within the kinase binding pocket, although this may simultaneously increase susceptibility to P-gp-mediated efflux. Conversely, the incorporation of polar or ionizable groups, including morpholine moieties or protonable amines, can improve aqueous solubility while increasing topological polar surface area, ultimately reducing passive diffusion across the BBB [62,82].

Furthermore, in multi-target inhibitors, structural extensions designed to access additional subpockets (e.g., the DFG-out region) often increase molecular size and rigidity, which can further compromise CNS penetration. Experimental SAR studies have demonstrated that even fine-tuning specific regions, such as aniline substituents in quinazoline-based inhibitors or the positioning of nitrogen atoms and hydrophobic groups within heterocyclic frameworks, can markedly influence efflux ratios and transporter selectivity. For example, in quinazolinamine derivatives, the positioning of nitrogen atoms within heterocyclic rings, together with the introduction of hydrophobic substituents (e.g., methyl, ethyl, n-propyl) or hydrogen bond donors/acceptors (e.g., methoxy, amine, pyridine), has been shown to strongly modulate inhibitory activity toward both P-gp and BCRP [83]. Similarly, studies on propafenone analogues have highlighted that nitrogen basicity, substituent flexibility, and lipophilicity (logP) are key determinants of transporter selectivity, with P-gp activity being particularly sensitive to changes in lipophilicity compared with BCRP [84].

Collectively, these findings underscore that parameters such as lipophilicity, nitrogen basicity and conformational flexibility differentially influence interactions with P-gp and BCRP, highlighting the intrinsic complexity of designing kinase inhibitors that balance efflux liability with target engagement. In this context, meticulous calibration of structural motifs is required to achieve an optimal compromise between potency and brain exposure. This balance is particularly critical in GB, where inadequate CNS penetration remains a major barrier to therapeutic efficacy, and where enhanced biochemical activity alone is insufficient without effective drug delivery to the tumor site.

### 5.5. Emerging Therapeutic Strategies: TKIs

Contemporary standard-of-care helps to control the primary tumor but rarely prevents relapses. Advances in next-generation sequencing (NGS) technologies have provided a deeper understanding of the genomic landscape of GB, leading to the development of targeted therapies to address the limitations of conventional treatments. Targeted therapy focuses on specific proteins that regulate the cell cycle, including growth, division, and dissemination. Within this framework, tyrosine kinase inhibitors (TKIs) have emerged as a primary area of interest due to their ability to target multiple pathways associated with GB. These inhibitors act upon specific molecular targets (protein kinases) that are either overactivated or mutated. By blocking these targets, TKIs aim to halt aberrant cellular signaling, thereby inhibiting or decelerating GB cell proliferation and the invasion of the surrounding microenvironment [2].

Although targeted therapy is a promising alternative due to its selectivity, it faces significant limitations. Primary among these is the BBB; many TKIs fail to penetrate the CNS or do so in insufficient quantities due to active efflux transport. This is mediated by pumps such as P-gp or ABC transporters, which actively remove the drug from the brain. Moreover, efficacy is compromised by intrinsic or acquired resistance, where a TKI may show initial effectiveness before the tumor adapts by activating bypass signaling pathways. Additionally, prolonged treatment often leads to off-target toxicity due to the inhibition of unintended kinases. Consequently, it is essential to advance research into drug design optimization and biodistribution to overcome the BBB and other obstacles that hinder the fight against the tumor and the subsequent recovery of the patient.

### 5.6. Receptor Tyrosine Kinase (RTK) Inhibitors for Glioblastoma

A comprehensive analysis of the human cancer kinome by Fleuren et al. [85] elucidated how dysregulation within the protein kinase superfamily drives malignant progression. Notably, their work highlights how kinome remodeling modulates drug sensitivity, particularly within receptor and non-receptor tyrosine kinase pathways activated via phosphorylation in GB.

The therapeutic utilization of kinase inhibitors in GB is predicated on the high prevalence of gene alterations affecting receptor tyrosine kinases (RTKs), including EGFR, PDGFR, MET or VEGF, which are pivotal drivers of tumor growth and survival. These receptors are frequently amplified or mutated, leading to constitutive, ligand-independent activation and sustained signaling through critical downstream pathways, such as PI3K/AKT/mTOR and RAS/RAF/MAPK/ERK. These pathways are involved in the regulation of cell proliferation, survival, differentiation and angiogenesis [33,86,87]. TKIs are designed to disrupt these processes by binding to the catalytic domain of the kinase, typically competing with ATP or targeting adjacent regulatory regions, thereby preventing receptor autophosphorylation and inhibiting downstream signal transduction [88].

**Figure 5:**
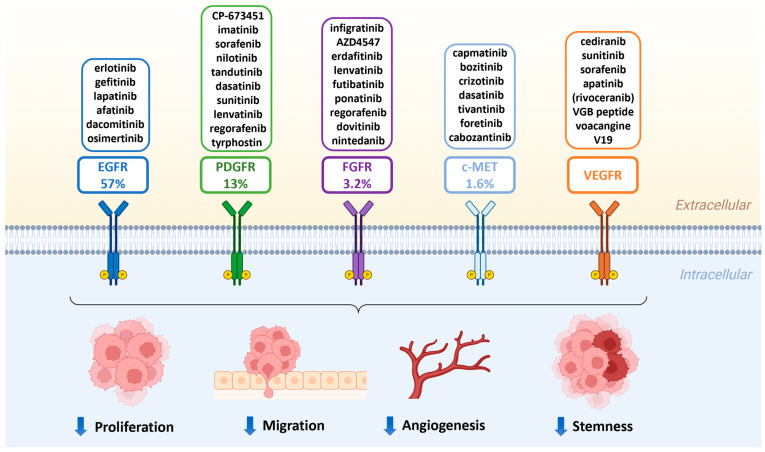
Overview of receptor tyrosine kinase inhibitors (RTKIs) for the treatment of GB. This figure represents the targeted inhibition of individual RTKs by their specific inhibitors. Blockade of these receptors prevents downstream signaling cascade activation, leading to a significant decrease in tumor cell proliferation, survival, invasion, stem-like properties, and angiogenesis, thereby targeting both glioblastoma progression and therapeutic resistance. Created in BioRender. Hijazi Vega, M. (2026) https://BioRender.com/vqyanyl (accessed on 16 July 2026).

Whole-exome sequencing (WES) of 291 GB patients revealed that 67% of cases harbor at least one alteration in RTKs. The mutational landscape is dominated by EGFR (57%), followed by PDGFR (13%), FGFR (3.2%), and c-MET (1.6%). Beyond surface receptors, downstream signaling is frequently compromised, with PTEN loss or mutation occurring in 41% of patients and PI3K mutations present in 25%, underlining the constitutive activation of the PI3K/AKT/mTOR axis in this malignancy [33,89]. Figure 5 provides an overview of the most relevant drugs for inhibiting the different signaling pathways that are disrupted in GB.

#### 5.6.1. Epidermal Growth Factor Receptor (EGFR)

The epidermal growth factor receptor (EGFR) is a transmembrane receptor tyrosine kinase composed of an extracellular ligand-binding domain, a transmembrane region and an intracellular kinase domain. Upon binding of ligands such as EGF or TGF-α, EGFR undergoes dimerization and autophosphorylation, triggering downstream signaling pathways that regulate cell proliferation and survival [90].

In GB, EGFR alterations represent the most frequent receptor tyrosine kinase abnormalities, occurring in approximately 45–57% of tumors [86]. These include gene amplification, mutations, rearrangements and alternative splicing events, all of which contribute to aberrant receptor activation and sustained oncogenic signaling. Among these, EGFR amplification is the most common and is strongly associated with aggressive tumor behavior, particularly in primary GB [91,92,93].

Particularly noteworthy is the EGFRvIII mutant variant, generated through an in-frame deletion of exons 2–7, which produces a constitutively active receptor independent of ligand binding. This alteration is present in around 20% of GBs and frequently co-occurs with EGFR amplification, further enhancing aberrant pathway activation [94,95]. In addition, dysregulation of other ERBB family members, including ERBB2 (HER2), has also been reported in a subset of tumors, contributing to the complexity and redundancy of oncogenic signaling networks in GB [96,97]. Despite the high prevalence and biological relevance of EGFR alterations, targeting this pathway has shown limited clinical success, mainly owing to tumor heterogeneity, adaptive resistance mechanisms and insufficient CNS exposure.

In this context, EGFR-targeted therapeutic strategies have primarily focused on two main classes of agents: TKIs, which bind to the intracellular catalytic domain, and monoclonal antibodies that target the extracellular receptor domain. From a medicinal chemistry perspective, most EGFR TKIs are built around ATP-mimetic heterocyclic scaffolds, particularly the 4-anilinoquinazoline core, which acts as a hinge-binding motif. This scaffold forms key hydrogen bonds with the kinase hinge region, while the anilino substituent extends into adjacent hydrophobic pockets, enabling fine-tuning of potency and selectivity through SAR optimization [98,99].

Accordingly, first-generation EGFR inhibitors such as erlotinib, gefitinib and lapatinib were designed as reversible ATP-competitive inhibitors to occupy the ATP-binding pocket of the kinase domain [100,101,102]. Structurally, they share a quinazoline core that anchors the molecule to the hinge region via hydrogen bonding, while hydrophobic substituents, such as phenyl rings, interact with the back pocket of the enzyme. Erlotinib exemplifies this design, combining a polar quinazoline nucleus with hydrophobic aromatic extensions that stabilize binding through apolar interactions [52]. It blocks cancer cell proliferation and cell cycle and inhibits downstream EGFR signal transduction [88]. Gefitinib, although structurally similar, incorporates a morpholine moiety that improves aqueous solubility and modulates pharmacokinetic properties, while halogen substitution enhances hydrophobic pocket interactions [62]. Despite strong preclinical activity and effective inhibition of EGFR-driven signaling pathways (including PI3K/AKT and MAPK), these agents showed limited clinical benefit in GB, likely due to intrinsic resistance mechanisms and insufficient brain exposure.

To address the limitations associated with reversible inhibition, second-generation inhibitors, including afatinib and dacomitinib, were developed to establish irreversible covalent interactions with the kinase domain [103,104,105]. Chemically, these compounds retain the quinazoline scaffold but incorporate electrophilic groups capable of undergoing Michael addition with conserved cysteine residues (e.g., Cys797 in EGFR; Cys805 in HER2, and Cys803 in HER4) [88]. This covalent binding enhances potency and prolongs target inhibition, and also enables activity against mutant forms such as EGFRvIII. Afatinib, for example, is another inhibitor that is taken orally and irreversibly inhibits multiple members of the ERBB family (EGFR, EGFRvIII, ERBB3 and ERBB4) [106,107], reflecting a broader inhibitory profile. Although these agents demonstrated improved activity in preclinical GB models, clinical outcomes remained modest, with limited progression-free survival benefits and concerns regarding toxicity [108]. Dacomitinib, an orally bioavailable pan-EGFR inhibitor, selectively targets EGFR, ERBB2, and ERBB4. Preclinical studies, encompassing both in vitro assays and in vivo animal models, have demonstrated its robust efficacy against EGFR-mutant lineages and lung cancer cells exhibiting gefitinib resistance [109].

More recently, third-generation inhibitors, particularly osimertinib, have represented further refinement in EGFR-targeted therapy by combining irreversible binding with increased selectivity for mutant EGFR variants. Osimertinib is specifically optimized to target resistance-associated mutations (e.g., T790M, a common mutation in non-small-cell lung cancer; NSCLC) while maintaining activity against EGFRvIII [110]. From a chemical standpoint, it incorporates structural features that enhance both selectivity and pharmacokinetic performance, including improved BBB penetration [111]. Notably, osimertinib achieves high brain-to-plasma ratios and effectively inhibits downstream signaling pathways in GB models, leading to reduced proliferation, invasion, and tumor growth. These properties make it one of the most promising EGFR inhibitors for CNS malignancies, although clinical validation in GB is still ongoing [112,113,114].

Collectively, the therapeutic landscape of EGFR inhibition has evolved through successive generations of inhibitors with increasing efficacy. For instance, the second-generation covalent inhibitor afatinib demonstrated a statistically superior progression-free survival (PFS) compared with the first-generation gefitinib (11.0 vs. 10.9 months) in a Phase IIB clinical trial [115]. More recently, the third-generation inhibitor osimertinib established a new clinical benchmark, yielding a median PFS of 18.9 months, nearly doubling the 10.2 months observed with first-generation agents such as erlotinib or gefitinib [116]. Nonetheless, despite these advances, effective EGFR-targeted therapy in GB continues to face major challenges arising from tumor heterogeneity, adaptive resistance pathways and the persistent obstacle of achieving adequate CNS drug delivery.

#### 5.6.2. Platelet-Derived Growth Factor Receptor (PDGFR)

Platelet-derived growth factor receptors (PDGFRs) are transmembrane receptor tyrosine kinases that play essential roles in normal cellular physiology, particularly during embryonic development, tissue repair, vascular maturation and angiogenesis [117]. Upon binding to platelet-derived growth factor (PDGF) ligands, PDGFR monomers undergo dimerization and subsequent intracellular autophosphorylation, triggering downstream signaling cascades including PI3K/AKT, RAS/MAPK, JAK/STAT and phospholipase Cγ pathways. Through these signaling networks, PDGFR regulates key cellular processes such as proliferation, migration, survival and differentiation [118,119]. In physiological settings, this signaling axis is tightly regulated; however, its dysregulation has been implicated in multiple malignancies, including GB, where it contributes to tumor growth, angiogenesis and resistance to apoptosis [88].

In GB, aberrant PDGFR signaling arises through receptor amplification, activating mutations and ligand-dependent autocrine activation. Both PDGFR isoforms (PDGFRα and PDGFRβ), together with all four PDGF ligands (PDGF-A, PDGF-B, PDGF-C and PDGF-D), have been detected in GB, supporting the existence of sustained autocrine and paracrine signaling loops that promote tumor progression [120]. Among these alterations, PDGFRα amplification represents the most frequent event, occurring in approximately 10–15% of GB cases [93]. This alteration is particularly enriched in the proneural molecular subtype and is frequently associated with IDH-mutant gliomas, in contrast to the classical subtype, which is more commonly characterized by EGFR amplification [91]. Notably, nearly half of PDGFRα-amplified tumors also harbor concurrent alterations in other receptor tyrosine kinases, particularly EGFR or MET, reflecting the extensive signaling redundancy that characterizes GB [121].

Beyond gene amplification, several activating PDGFRα mutations have also been identified as oncogenic drivers. For instance, the Δ8,9 extracellular deletion and the V536E transmembrane substitution promote constitutive receptor activation and glioma proliferation through persistent ERK signaling [122,123]. In parallel, PDGFRβ further contributes to GB pathogenesis through its enrichment in GSCs and tumor-associated endothelium, where it facilitates vascular remodeling and invasiveness [124]. Together, these multifaceted alterations establish PDGFR signaling as a pivotal driver of glioblastoma progression and reinforce its relevance as a therapeutic target, particularly within precision medicine strategies aimed at molecularly defined patient subsets [117].

Consequently, substantial efforts have focused on the development of therapeutic agents targeting PDGFR signaling in GB. From a medicinal chemistry perspective, most PDGFR inhibitors belong to the broader class of ATP-competitive tyrosine kinase inhibitors and are generally constructed around heterocyclic scaffolds capable of mimicking ATP while establishing key hydrogen-bond interactions with the kinase hinge region. Both selective inhibitors and multitarget kinase inhibitors have been investigated, reflecting the complexity and redundancy of PDGFR-associated signaling networks in GB.

Among the most selective compounds described in preclinical models is CP-673451, a small-molecule TKI with high selectivity for PDGFRβ, with an IC_50_ of approximately 1 nM and nearly 450-fold selectivity over other PDGFR family members [125,126]. Interestingly, beyond its antiproliferative effects, CP-673451 has been shown to induce neurite-like differentiation in multiple GB cell lines, including U87, U251 and LN229, suggesting a potential role beyond cytotoxicity, promoting a more differentiated cellular phenotype [127]. Furthermore, in vivo, it enhances the antitumor efficacy of TMZ, significantly reducing tumor volume in xenograft models [128]. Pharmacokinetic studies in rat model additionally revealed that oral administration at 50 mg/kg achieves plasma concentrations above the effective threshold (EC_50_) for several hours (≈4 h), supporting its potential as a bioavailable PDGFR-targeted agent [125].

In contrast to highly selective compounds, several multi-target TKIs such as imatinib and sorafenib illustrate the challenges of targeting PDGFR within a complex signaling network. Imatinib, originally developed to target ABL, also inhibits PDGFR and KIT through a type II binding mode, stabilizing the inactive conformation of the kinase domain [129]. Chemically, it is characterized by a flexible structure containing multiple aromatic rings and hydrogen-bond-forming functionalities that allow interaction with both the ATP-binding site and adjacent hydrophobic pockets. Despite its success in hematological malignancies such as chronic myeloid leukemia [130], imatinib has shown limited efficacy in GB clinical trials, either as monotherapy [131] or in combination with hydroxyurea [132]. This lack of activity is partly attributed to compensatory signaling through parallel RTKs such as ERBB3 and IGF1R, underscoring the importance of pathway redundancy in GB [132]. Similarly, sorafenib, a multi-kinase inhibitor targeting PDGFR, VEGFR, RAF, FLT3, and c-KIT, failed to demonstrate clinical benefit in phase III trials, despite its broad inhibitory profile [133].

Other PDGFR-targeting agents, including nilotinib or tandutinib, have also been evaluated in clinical settings, but with limited success. Nilotinib (AMN107), a second-generation analogue of imatinib with improved potency and pharmacokinetics, did not demonstrate meaningful activity in PDGFR-amplified gliomas [134]. Similarly, tandutinib (MLN518), a PDGFRβ inhibitor, did not improve outcomes in recurrent GB [135] and was associated with increased toxicity when combined with bevacizumab [136]. These results highlight that simply increasing potency or selectivity at the molecular level does not necessarily translate into clinical efficacy in GB. In parallel, several multi-kinase inhibitors, including dasatinib, sunitinib, lenvatinib and regorafenib, have been explored in GB. Among them, regorafenib represents the most clinically relevant example of a multitarget kinase inhibitor achieving a measurable survival benefit in recurrent GB. Chemically, regorafenib is an oral small-molecule inhibitor targeting VEGFR, PDGFR, RAF, FGFR and additional kinases involved in angiogenesis, tumor proliferation and microenvironment remodeling. This broad inhibitory spectrum is particularly advantageous in GB, where extensive molecular heterogeneity and signaling redundancy frequently limit the efficacy of highly selective agents [80,137]. The REGOMA trial demonstrated improved overall survival compared with lomustine in recurrent GB patients, and subsequent studies confirmed its reproducible activity, even in heavily pretreated populations [61,137,138]. Nevertheless, clinical responses remain highly variable, suggesting that efficacy is strongly influenced by tumor molecular context, pharmacokinetic variability and adaptive resistance mechanisms. Moreover, the broad polypharmacology of regorafenib is associated with significant systemic toxicity, often requiring dose reductions, thereby illustrating the delicate balance between therapeutic efficacy and tolerability in multitarget kinase inhibition [137,139]. Preclinical compounds such as tyrphostins (AG1296, AG1433) further demonstrate that PDGFR inhibition can suppress tumor growth and enhance radiosensitivity, yet their clinical development remains limited [140].

Overall, the pharmacological targeting of PDGFR in GB illustrates a key challenge in kinase inhibitor design: achieving sufficient pathway suppression in a highly redundant signaling environment while maintaining adequate brain exposure. These findings suggest that future strategies should move beyond single-agent PDGFR inhibition and instead focus on rational combination therapies or network-level targeting approaches to overcome resistance mechanisms inherent to GB biology.

#### 5.6.3. Fibroblast Growth Factor Receptor (FGFR)

Fibroblast growth factor receptors (FGFRs) are transmembrane receptor tyrosine kinases involved in the regulation of cell proliferation, differentiation, migration, angiogenesis and tissue repair. Upon ligand binding, FGFRs undergo receptor dimerization and autophosphorylation, activating downstream pathways such as RAS/MAPK, PI3K/AKT and PLCγ signaling [141]. Compared with EGFR and PDGFR alterations, FGFR aberrations in GB are relatively infrequent; however, they are biologically and clinically significant in defined molecular subsets. Among these, the most well-characterized alteration is the FGFR3-TACC3 fusion, generated by chromosomal rearrangement between the FGFR3 gene and the transforming acidic coiled-coil-containing gene 3 (TACC3) gene, which occurs in approximately 3% of GB cases [86,142]. Structurally, this fusion links the intracellular tyrosine kinase domain of FGFR3 to the coiled-coil domain of TACC3, promoting constitutive receptor dimerization and ligand-independent kinase activation. Functional studies demonstrated that FGFR3-TACC3 fusion proteins possess oncogenic activity capable of transforming astrocytes into glioma cells in murine brain models, supporting their direct role in GB pathogenesis [141,142,143,144,145]. In parallel, FGFR alterations are often associated with aggressive tumor behavior and poor prognosis, further underscoring their clinical relevance.

Beyond fusion events, heterogeneous expression of FGFR1-4 has been reported across GB subtypes, with FGFR1 particularly linked to glioma stem cell maintenance, invasion and radioresistance [146,147,148]. These observations suggest that FGFR signaling contributes not only to tumor proliferation but also to therapeutic resistance and recurrence. Furthermore, GB frequently exhibits simultaneous alterations in multiple receptor tyrosine kinases, highlighting the molecular heterogeneity of the disease and the existence of compensatory signaling networks [87].

Recent evidence also indicates that FGFR signaling may act as an adaptive bypass mechanism during targeted therapy. GB cells are capable of evading EGFR or MET inhibition through FGFR-SPRY2-mediated signaling, suggesting that FGFR activation may contribute to resistance against other RTK inhibitors [149]. Furthermore, metabolic studies have demonstrated that FGFR3-TACC3-positive tumors exhibit a marked reliance on mitochondrial oxidative phosphorylation, thereby revealing a distinct metabolic vulnerability associated with this molecular subgroup [145]. Collectively, these findings position FGFR alterations as infrequent but highly relevant therapeutic targets in glioblastoma biology.

In response to these discoveries, several FGFR-targeted therapeutic strategies have been developed, primarily based on ATP-competitive small-molecule inhibitors designed to bind the conserved kinase domain via heteroaromatic scaffolds that interact with the ATP-binding hinge region [147]. Among the most extensively studied compounds are infigratinib (BGJ398) and fexagratinib (AZD4547), both of which act as selective pan-FGFR inhibitors with higher potency against FGFR1–3. Preclinical studies demonstrated significant inhibition of tumor proliferation and tumor growth in FGFR-altered models. Clinically, infigratinib induced partial responses or stable disease in approximately one-third of recurrent GB patients carrying FGFR alterations, supporting further biomarker-driven studies [150,151].

Similarly, erdafitinib (JNJ-42756493), an orally active pan-FGFR inhibitor targeting FGFR1–4, has also shown promising results [152,153]. In preclinical glioma models harboring FGFR3-TACC3 rearrangements, erdafitinib suppressed tumor cell proliferation and reduced tumor growth in vivo. Early clinical observations additionally reported disease stabilization and minor responses in FGFR3-TACC3-positive glioma patients, supporting the therapeutic relevance of FGFR inhibition in this subgroup [144,154].

In addition to selective inhibitors, several multitarget kinase inhibitors, including lenvatinib, futibatinib, ponatinib, regorafenib, dovitinib and nintedanib, also inhibit FGFR signaling alongside VEGFR, PDGFR and other RTKs. Although this broader activity may be advantageous in the highly heterogeneous signaling environment of GB, it is frequently associated with increased toxicity and limited selectivity [33,155,156,157,158].

Overall, the therapeutic potential of FGFR inhibitors in GB appears largely restricted to a small molecularly defined subset of patients, particularly those harboring FGFR3-TACC3 fusions. Consequently, these observations highlight the critical importance of molecular stratification and the potential value of combination therapeutic strategies to maximize the clinical efficacy of FGFR-targeted approaches in GB.

#### 5.6.4. Mesenchymal Epithelial Transition Factor Receptor (MET)

The mesenchymal–epithelial transition factor receptor (MET), also known as the hepatocyte growth factor receptor (HGFR), is a transmembrane receptor tyrosine kinase that is activated by hepatocyte growth factor (HGF). Under physiological conditions, the HGF/MET signaling axis regulates essential biological processes, including embryonic development, tissue regeneration, cell proliferation, migration, and survival. However, in GB, aberrant activation of MET signaling contributes to tumor growth, invasion, angiogenesis, and therapeutic resistance. In this context, elevated MET expression has been consistently associated with a poor prognosis and shorter patient survival [159,160,161].

In GB, MET dysregulation occurs through multiple and often overlapping molecular mechanisms, including receptor overexpression, gene amplification, activating mutations, exon skipping events, and oncogenic fusion [159]. Specifically, MET overexpression is detected in approximately 20–30% of high-grade gliomas [162,163], whereas MET amplification occurs in nearly 4% of primary GB cases [94]. In addition, constitutively active MET variants have been identified, including the METΔ7-8 deletion mutant, which lacks exons 7 and 8 and generates a ligand-independent receptor with predominant cytosolic localization [164]. Another clinically relevant alteration is MET exon 14 skipping (METex14), which prevents receptor degradation by deleting the intracellular juxtamembrane regulatory domain. METex14 often co-occurs with the PTPRZ1-MET (ZM) fusion, detected in approximately 15% of secondary GBs and associated with particularly poor prognosis [165].

Furthermore, experimental evidence from murine models has reinforced the oncogenic role of MET signaling in glioma genesis. Studies have demonstrated that activation of the HGF/MET axis, especially in combination with tumor suppressor loss such as p53 deficiency [166] or alterations in Ink4b/Arf pathways, drives glioma initiation and progression [167]. Moreover, MET-driven tumors frequently express glioma stem cell markers, highlighting the role of MET signaling in maintaining stemness and tumor aggressiveness. Importantly, MET activation has also been implicated in adaptive resistance mechanisms, particularly following anti-VEGF or anti-EGFR therapies, where compensatory MET signaling promotes a more invasive tumor phenotype [161,168,169].

Given this biological relevance, considerable efforts have been devoted to the development of MET-targeted therapies in GB, ranging from selective ATP-competitive inhibitors to broad-spectrum multitarget kinase inhibitors. From a structural perspective, most MET inhibitors are designed around heterocyclic scaffolds that mimic ATP and interact with the kinase hinge region, thereby blocking downstream signaling. Among the most selective agents, capmatinib (INC280) stands out as a highly potent MET inhibitor with an IC_50_ of approximately 0.13 nM [170]. Preclinical studies have demonstrated its strong activity in MET-amplified, MET-overexpressing, and HGF-dependent tumor models, where it effectively suppresses MET signaling in vitro and in vivo [171,172,173]. Building on these findings, capmatinib has been evaluated in clinical settings, including in combination with bevacizumab for recurrent GB (NCT02386826). However, subsequent clinical data revealed that combination therapy with buparlisib in MET-amplified tumors failed to produce meaningful responses, thereby underscoring the intrinsic therapeutic resistance associated with this molecular subtype [174].

In parallel, alternative strategies have focused on improving BBB penetration and overcoming resistance mechanisms. In this context, bozitinib (PLB-1001) was rationally designed to interact not only with the ATP-binding pocket of MET but also with additional interaction sites within the kinase domain, increasing binding affinity and inhibitory potency relative to crizotinib [165]. Importantly, PLB-1001 demonstrated effective BBB permeability and superior activity against MET-driven GB xenograft models (positive phase I study) (NCT02978261). Early clinical studies reported partial responses in patients harboring METex14 alterations or PTPRZ1-MET fusions, supporting MET as a therapeutically actionable target in molecularly selected GB subgroups.

Similarly, crizotinib, originally developed as a dual MET/ALK inhibitor, also showed selective activity against MET-positive glioma stem cells and reduced tumor growth in vivo [175]. Crizotinib is currently under clinical investigation for GB in combination with temozolomide and radiotherapy (NCT02270034) [176]; however, its evaluation alongside dasatinib in pediatric high-grade and diffuse pontine gliomas (NCT01644773) revealed poor tolerability and minimal clinical activity [177]. Similarly, tivantinib reduced proliferation and colony formation in U251 and T98MG GB cell lines at higher concentrations, particularly when combined with PI3K (LY294002) or mTOR (rapamycin) inhibitors, highlighting the importance of pathway co-targeting in MET-driven tumors [178].

In addition to selective inhibitors, several multitarget TKIs have been explored for their anti-MET activity. For instance, foretinib, which targets MET, VEGFR, PDGFR, AXL, RON and Tie-2, has been shown in preclinical GB models to induce G2/M cell cycle arrest, trigger mitochondrial apoptosis and reduce tumor invasiveness through downregulation of MMP2, uPA/uPAR signaling and epithelial–mesenchymal transition-associated genes [179]. However, despite these promising preclinical effects, its limited penetration into the brain significantly restricts its therapeutic potential [179]. Likewise, cabozantinib, another multitarget inhibitor acting against MET, AXL and VEGFR, has demonstrated antiangiogenic and antitumor activity in xenograft models and synergistic effects with radiotherapy in GB cell lines. Nevertheless, clinical trials (phase II studies) in recurrent GB have shown only modest efficacy and significant toxicity, failing to meet predefined efficacy endpoints [180,181].

Overall, the accumulated evidence indicates that MET inhibition alone is rarely sufficient to achieve durable therapeutic responses in GB, primarily due to extensive signaling redundancy and rapid adaptive rewiring of oncogenic networks. In particular, resistance to MET-targeted therapies is frequently mediated by compensatory activation of alternative pathways, including EGFR, VEGF, FGFR1, mTOR, STAT3 and COX-2 signaling. Consequently, current therapeutic strategies are increasingly shifting towards rational combination approaches, especially dual inhibition of MET with EGFR or VEGF pathways, which have demonstrated enhanced antitumor efficacy in both preclinical and translational studies [161,168,169,182].

#### 5.6.5. Vascular Endothelial Growth Factor Receptor (VEGFR)

Vascular endothelial growth factor receptors (VEGFRs) are transmembrane receptor tyrosine kinases that play a central role in the regulation of vasculogenesis, angiogenesis, and vascular permeability. The VEGFR family comprises three major members, VEGFR1, VEGFR2, and VEGFR3, where VEGFR1 and VEGFR2 are primarily responsible for physiological and pathological angiogenesis, whereas VEGFR3 regulates lymphangiogenesis and embryonic vascular development [183]. In GB, VEGFR signaling is markedly upregulated and closely associated with tumor grade, vascular proliferation, and the development of an immunosuppressive tumor microenvironment [184,185].

In this context, angiogenesis represents a defining hallmark of GB progression and is predominantly driven by hypoxia-induced VEGF overexpression [185]. Under hypoxic conditions, the stabilization of hypoxia-inducible factors (HIF-1α and HIF-1β) promotes transcriptional activation of VEGF through binding to hypoxia-response elements within the VEGF promoter region [186]. Subsequently, increased VEGF secretion activates VEGFR signaling in endothelial cells, thereby triggering proliferation, migration, vascular permeability, and neovascularization. In parallel, additional proangiogenic mediators, including PDGF, FGF, ANG-1/2, DLL4, IL-8, and SDF-1, further amplify this complex vascular remodeling program [187].

Unlike EGFR or MET, activating mutations in VEGFR are relatively uncommon in GB; instead, pathway dysregulation predominantly results from VEGF overexpression together with VEGFR2 upregulation within tumor-associated endothelial cells. In particular, VEGFR2 is highly expressed in GB vasculature and constitutes one of the principal drivers of abnormal tumor angiogenesis [187]. Importantly, early experimental studies demonstrated that suppression of VEGF expression significantly reduced vascular formation and inhibited glioma growth, thereby establishing the VEGF/VEGFR axis as a critical therapeutic target in GB [188].

Given this strong biological rationale, extensive efforts have been devoted to targeting VEGFR signaling through monoclonal antibodies (e.g., bevacizumab), peptide-based inhibitors (e.g., aflibercept) and ATP-competitive tyrosine kinase inhibitors. Among small-molecule inhibitors, cediranib represents one of the most extensively characterized VEGFR inhibitors in GB. Cediranib is an ATP-competitive inhibitor with high affinity for VEGFR2, while also inhibiting PDGFRβ and c-KIT [189]. Structurally, cediranib belongs to the class of orally bioavailable heteroaromatic kinase inhibitors designed to occupy the ATP-binding cleft of VEGFR kinases, thereby preventing receptor autophosphorylation and downstream angiogenic signaling. Despite preclinical evidence demonstrating that the VEGFR-2 inhibitor cediranib effectively inhibited neovascularization and induced regression of established tumor vessels, its clinical translation has been paradoxical. Specifically, although cediranib initially promotes structural and functional normalization of the tumor vasculature, improving perfusion and drug delivery, this physiological stabilization does not translate into sustained therapeutic advantage or survival benefit. Accordingly, a Phase III clinical trial evaluating cediranib either as a monotherapy or in combination with lomustine failed to improve progression-free survival [190]. Similarly, in a Phase II study (NCT00305656), early radiographic responses were observed but did not result in significant overall survival benefits in patients with recurrent glioblastoma [191]. This apparent discrepancy between biological activity and clinical outcome highlights the complexity of angiogenesis-targeted therapies in GB.

In addition to selective VEGFR inhibitors, several multitarget tyrosine kinase inhibitors such as sunitinib and sorafenib have been developed to simultaneously inhibit angiogenesis and tumor cell proliferation by targeting VEGFR, PDGFR, KIT, FLT3 and RAF kinases [192,193]. Nevertheless, despite encouraging preclinical antiangiogenic effects, clinical trials in recurrent GB have shown limited efficacy and no significant survival advantage [194,195].

More recently, the development of next-generation VEGFR inhibitors has focused on improving selectivity and pharmacological potency. In this context, apatinib (rivoceranib) is a highly selective VEGFR2 inhibitor with an IC50 of approximately 16 nM [196]. Its molecular design favors strong interactions with the VEGFR2 ATP-binding pocket, leading to potent inhibition of VEGF-mediated angiogenic signaling. In recurrent GB patients previously treated with standard chemoradiotherapy, apatinib has shown modest improvements in progression-free survival, although its clinical use is frequently limited by toxicity, including thrombocytopenia and hand-foot syndrome [197].

In parallel, alternative therapeutic modalities such as peptide-based VEGFR inhibitors have also been investigated to enhance target specificity. For example, VGB, a synthetic peptide derived from VEGFA interaction motifs, was rationally designed to bind both VEGFR1 and VEGFR2 and has demonstrated inhibition of proliferation in VEGFR2-overexpressing U87 GB cells with submicromolar potency (IC50 ≈ 0.92 μM), thereby supporting the therapeutic relevance of VEGFR2 blockade in GB [198]. Likewise, small molecules such as the indole alkaloid voacangine and its synthetic analogue V19 have demonstrated antiangiogenic activity through inhibition of VEGFR2 phosphorylation and suppression of HIF-1α/VEGF signaling, without inducing major cytotoxicity in preclinical GB models [199,200].

Despite these advances, the clinical efficacy of VEGFR-targeted therapies in GB remains limited by multiple biological and pharmacokinetic barriers. In particular, many VEGFR inhibitors are substrates of efflux transporters such as P-gp and BCRP, which significantly restrict their penetration into the central nervous system [201]. This limitation affects a wide range of compounds, including cediranib, pazopanib, sunitinib, sorafenib, regorafenib, tandutinib, axitinib, and vatalanib [33,39,202]. In addition, antiangiogenic therapies frequently trigger compensatory activation of alternative pathways, particularly MET signaling, promoting a more invasive tumor phenotype. Consequently, these adaptive resistance mechanisms have shifted current therapeutic strategies toward rational combination approaches, especially those integrating VEGFR inhibition with MET blockade, immune checkpoint inhibition or radiotherapy, in order to overcome signaling redundancy and improve therapeutic durability in glioblastoma [169,203].

Given that RTK dysregulation constitutes a core driver of GB pathogenesis, establishing a direct link between genomic profiling and targeted therapy is essential for modern neuro-oncology. In this sense, Table 1 presents a compilation of major genetic RTK alterations in GB, systematically defining their clinical relevance, functional impact, and downstream network effects. Complementing this genomic framework, Table 2 shows the specific kinase inhibitors developed to exploit these therapeutic vulnerabilities, their targeted signaling pathways and their translational status across documented clinical trials. Similarly, Table 3 highlights a list of compounds used in experimental preclinical studies separated from the formal clinical trials.

Beyond the canonical pathway alterations in RTK, another small but highly actionable subset of GB harbors low-frequency molecular vulnerabilities, specifically BRAF V600E mutations and NTRK gene fusions. Although rare in adult diffuse gliomas, these alterations represent major therapeutic targets that bypass standard genotoxic resistance. For instance, dual BRAF/MEK inhibition has demonstrated significant clinical efficacy in BRAF-mutant high-grade gliomas, offering a precise alternative to conventional treatment [204]. Similarly, highly selective TRK inhibitors have shown robust and durable intracranial activity in tumors harboring NTRK fusions [205].

**Table 1:** Major Genetic RTK Alterations in GB.

RTK Pathway	Genetic Alteration in GB	Frequency in GB	Biological Function and Clinical Relevance	Key Downstream Pathways Affected	Reference
EGFR	EGFR amplification	~45–57% of GB	Most frequent RTK alteration in GB. Promotes constitutive proliferation, survival, invasion and resistance to apoptosis. Strongly associated with primary/classical GB subtype and aggressive tumor behavior.	PI3K/AKT/mTOR; RAS/RAF/MAPK/ERK	[86,93]
EGFR	EGFRvIII deletion mutant (exons 2–7 deletion)	~20% of GB	Ligand-independent constitutively active receptor. Enhances tumor growth, stemness, invasion and therapeutic resistance. Frequently coexists with EGFR amplification.	PI3K/AKT; STAT3; MAPK	[94,95,206]
EGFR/ERBB family	ERBB2 (HER2) alterations	Low frequency (<5%)	Contributes to RTK signaling redundancy and compensatory activation, reducing efficacy of EGFR-targeted therapies.	PI3K/AKT; MAPK	[96,97]
PDGFRα	PDGFRα amplification	~10–15% of GB	Common in proneural and IDH-mutant GB. Promotes proliferation, angiogenesis and glioma stem-cell maintenance. Frequently coexists with EGFR or MET alterations.	PI3K/AKT; RAS/MAPK; JAK/STAT	[91,93]
PDGFRα	Δ8,9 extracellular deletion mutant	Rare	Generates constitutive receptor activation and uncontrolled proliferative signaling through ERK activation.	MAPK/ERK	[123]
PDGFRα	V536E transmembrane substitution	Rare	Constitutively activates PDGFRα signaling and enhances glioma proliferation and oncogenicity.	ERK signaling	[122]
PDGFRβ	Overexpression in glioma stem cells and tumor vasculature	Common endothelial enrichment	Supports vascular remodeling, invasiveness and tumor microenvironment maintenance. Associated with aggressive angiogenic phenotype.	PI3K/AKT; PLCγ	[124]
FGFR3	FGFR3-TACC3 fusion	~3% of GB	Constitutively active fusion kinase generated by chromosomal rearrangement. Promotes ligand-independent signaling, tumor growth and metabolic rewiring. Defines a molecularly actionable GB subgroup.	MAPK; PI3K/AKT; mitochondrial OXPHOS	[86,142,143,145]
FGFR1	FGFR1 overexpression	Variable	Associated with glioma stem-cell maintenance, invasion, recurrence and radioresistance.	MAPK; PI3K/AKT	[146,147,148]
MET	MET overexpression	~20–30% of high-grade gliomas	Correlates with poor prognosis, increased invasion, angiogenesis and stemness phenotype. Promotes aggressive mesenchymal transition.	PI3K/AKT; MAPK	[159,162,163]
MET	MET amplification	~4% of primary GB	Leads to constitutive activation of MET signaling and resistance to anti-VEGF therapies.	PI3K/AKT; MAPK	[94]
MET	METΔ7–8 deletion mutant	Rare	Ligand-independent constitutively active receptor variant with enhanced oncogenic signaling.	MAPK; PI3K/AKT	[164]
MET	MET exon 14 skipping (METex14) and PTPRZ1-MET fusion (ZM fusion)	~15% of secondary GB	Prevents receptor degradation and sustains MET activation. Frequently associated with aggressive disease and therapeutic resistance.	MET survival signaling	[165]
VEGF/VEGFR axis	VEGF overexpression	Very common in GB	Central driver of angiogenesis under hypoxia. Promotes abnormal vessel formation, vascular permeability and tumor progression. Strongly associated with tumor grade.	VEGFR2 signaling; HIF-1α axis	[185]
VEGFR2	VEGFR2 upregulation in tumor endothelium	Highly expressed in GB vasculature	Principal mediator of tumor angiogenesis and endothelial proliferation. Major therapeutic target for antiangiogenic therapies.	VEGF/VEGFR2 signaling	[169]

**Table 2:** Kinase inhibitors included in clinical trials related to GB.

Kinase Inhibitor	Molecular Target/Pathway	Trial Phase & NCT ID	Patient Population & Sample Size (N)	Biomarker Selection Criteria	Main Endpoint	Efficacy & Clinical Outcomes	Toxicity & Safety Profile	Ref.
Erlotinib	EGFR (ErbB1)	Phase II NCT: N/A	Recurrent GB (N = 110)	Unselected	6-month Progression-Free Survival (PFS-6)	PFS-6: 11.4% (vs. 24% in control arm) Median Overall Survival (OS): ~6 months Modest single-agent activity with limited BBB penetration	General: Well tolerated Frequent Adverse Events (AEs): Skin toxicity was the most frequent effect	[102]
Gefitinib	EGFR	Phase II NCT: N/A	Newly diagnosed GB/grade 4 astrocytoma, stable or improved after radiotherapy (RT) (N = 98 enrolled, 96 evaluable)	EGFR amplification/mutation assessed by FISH and IHC (when tissue available)	1-year OS from initial surgery (specifically 52-week survival after biopsy/surgery)	1-year OS: 54.2% (vs. 48.9% in historical controls, *p* = NS) 1-year post-RT PFS: 16.7% (vs. 30.3% in historical controls) Outcomes were not significantly different from controls and not affected by EGFR status	Frequent AEs: Rash (62%), loose stools (58%), and fatigue (41%)	[100]
Lapatinib	EGFR/HER2	Phase I/II NCT: N/A	Recurrent GB (N = 24 total, Ph II: 17 non-EIAEDs, Ph I: 7 on EIAEDs)	Unselected (PTEN and EGFRvIII tested for exploratory correlation)	Determine response rate, pharmacokinetics, and recommended dose with Enzyme-Inducing Anti-Epileptic Drugs (EIAEDs)	Disease Control: Stable disease (SD) in 4/17 patients, progression in 13/17 patients No significant activity in GB, accrual stopped early due to lack of efficacy	Dose-Limiting Toxicity (DLT): None observed in Phase I Pharmacokinetics: Oral clearance was ~10-fold higher when co-administered with EIAEDs	[101]
Afatinib	EGFR, EGFRvIII, ERBB3, ERBB4	Phase I/Randomized Phase II NCT: N/A	Recurrent GB (N = 151 total, Ph I safety: 32, Ph II: 119)	EGFR-amplified or EGFRvIII positive	PFS-6 and Response Rate	PFS-6: 3% (afatinib), 10% (afatinib + temozolomide), and 23% (temozolomide alone) Objective Response: Partial response (PR) in 1, 2, and 4 patients, SD in 14, 14, and 21 patients, respectively Limited single-agent activity, median PFS was longer in EGFRvIII-positive tumors	General: Recommended Phase II Dose (RP2D) was 40 mg/day with temozolomide Frequent AEs: Diarrhea (71% mono, 82% combo) and rash (71% mono, 69% combo)	[108]
Nilotinib (AMN107)	PDGFR-α, BCR-ABL	Phase II NCT: N/A	Adults with recurrent GB (N = 34, 22 IDH-wt, 2 IDH-mut, 10 NOS)	PDGFR-α amplification or PDGFR-α overexpression	PFS-6, Safety, OS, and Objective Response Rate (ORR)	PFS-6 of 9% (3/34), PFS-12 of 6% (2/34), median PFS 1.3 months, median OS 6.6 months ORR 3% (1/34), 1 durable Complete Response (CR) >5 years in a PDGFR-α overexpressing tumor, 8 SD Overall modest activity	Severe Toxicity: 18% (6/34) experienced treatment-related Grade ≥3 AEs Mortality: No Grade 5 AEs reported, specific toxicities were not detailed	[134]
Regorafenib	VEGFR1–3, PDGFR, FGFR, RAF-1, KIT, BRAF, RET	Phase II NCT02928575 (REGOMA)	Recurrent GB (N = 119, 59 regorafenib vs. 60 lomustine)	Unselected (MGMT methylation and IDH status collected but not for inclusion)	PFS and OS at 6 months	Median OS 10.2 months, 1-year OS (OS-12) 43%, median PFS 2.3 months, PFS-6 18% PR in 7.4%, SD in 38.9% (Disease Control Rate: 46.3%) Encouraging efficacy in recurrent glioblastoma settings	Severe Toxicity: Grade 3 drug-related AEs in 18%, Grade 4 in 2%, no drug-related deaths Management: 37% required dose reduction, 6% discontinued Frequent AEs: Hand-foot skin reaction, hypertransaminasemia, rash	[207]
Infigratinib (BGJ398)	FGFR1–3	Phase I (Dose-escalation/expansion) NCT01004224	Adults with advanced solid tumors harboring FGFR alterations (N = 132)	FGFR3-TACC3 fusions or FGFR mutations	Maximum Tolerated Dose (MTD), RP2D, and dosing schedule	MTD/RP2D at 125 mg/day (3-weeks-on/1-week-off) 6 patients achieved PR, 1 unconfirmed PR 42 patients achieved stable diseases Clarified dosing parameters and showed stable disease in a subset of advanced solid tumors	Severe Toxicity: 99.2% experienced AEs (52.3% Grade 3/4), 13.6% discontinued, 1 treatment-related death. Frequent AEs: Hyperphosphatemia (74.2%), constipation (40.2%), appetite decrease (40.2%).	[150]
Capmatinib (INC280)	MET	Phase Ib/II NCT02386826	MET-altered GB (N = 43 total, Ph Ib combo with buparlisib: 33, Ph II mono: 10)	MET-amplified or high c-MET expression	Ph Ib: MTD/RP2D for combination with buparlisib. Ph II: Efficacy and safety (PFS-6 not assessed due to sample size)	Mono Efficacy: 0 PR/CR, SD in 3/10 (30%) lasting 16–20 weeks Combo Efficacy: Very limited activity, no RP2D declared due to low exposure/DDI Phase II halted early due to insufficient single-agent activity	Combo MTD: Capmatinib 300 mg BID + buparlisib 80 mg QD Severe Toxicity: Grade ≥3 AEs in 36.4% (combo) and 20.0% (mono). DLTs included nausea, personality changes, elevated transaminase. Frequent AEs (Mono): Headache (40%), constipation (30%), fatigue (30%), lipase increase (30%).	[173]
Bozitinib (PLB-1001)	MET	Phase I NCT02978261	Chemo-resistant glioma patients (N = N/A)	PTPRZ1-MET fusions or MET amplification	Safety and Efficacy parameters	Objective Response: Achieved PR in at least two patients with advanced GB Preclinical: Demonstrated remarkable potency in selectively inhibiting MET-altered cells Showed early signs of clinical activity in MET-altered gliomas	Side effects were described as rarely significant Specific adverse events, grades, and frequencies were not reported	[165]
Crizotinib + Dasatinib	Dasatinib: PDGFRA/B, Src, c-Kit Crizotinib: ALK, c-Met	Phase I NCT02270034	Children with recurrent High-Grade Glioma (HGG) or diffuse intrinsic pontine gliomas (DIPG) (N = 25 total, 11 with DIPG)	PDGFRA amplification/mutation or MET amplification	Establish safety, tolerability, and MTD of the combination	MTD Parameters: Dasatinib 50 mg/m^2^ QD + crizotinib 215 mg/m^2^ QD (BID dosing was poorly tolerated) No objective radiologic responses observed Durability: Only 2 patients remained on therapy for ≥6 months Overall clinical activity was minimal	DLTs & AEs: Combination was poorly tolerated, DLTs included diarrhea, fatigue, proteinuria, hyponatremia, rash, and Grade 4 neutropenia	[177]
Cediranib (Phase II)	VEGFR1–3, PDGFRβ, c-KIT	Phase II NCT00305656	Recurrent GB (N = 31)	Plasma and urinary biomarker evaluations	PFS-6	PFS-6 (APF6) was 25.8% Radiographic PR in 17/30 (56.7%) by 3D-MRI and 8/30 (27%) by 2D-MRI Allowed corticosteroid dose reduction in 10/15 and discontinuation in 5/15 patients Associated with encouraging radiographic responses and steroid-sparing effects	Severe Toxicity: Grade 3/4 events included fatigue (19.4%), hypertension (12.9%), and diarrhea (6.4%) 48.4% required dose reductions, 15 patients required temporary drug interruptions due to toxicity	[191]
Cediranib (Phase III)	VEGFR1–3, PDGFRβ, c-KIT	Phase III NCT00777153 (REGAL)	Adults with recurrent GB post-RT and temozolomide (N = 325 randomized, 131 cediranib, 129 cediranib + lomustine, 65 lomustine + placebo)	Unselected	PFS by blinded independent radiographic assessment	Median PFS was 92 days (mono), 125 days (combo), and 82 days (lomustine control). Median OS was 8.0, 9.4, and 9.8 months, respectively Failed to meet primary endpoint, PFS was not significantly improved with mono (HR 1.05) or combo (HR 0.76). No OS differences	Frequent AEs: Diarrhea was common (71% mono, 71% combo, 19% control) Combination increased hematologic toxicity, notably thrombocytopenia (38.3%) and neutropenia (20.3%), exacerbating lomustine-induced effects	[190]
Sunitinib	VEGFR1–3, PDGFR, c-KIT, RET	Phase II NCT00923117	Recurrent GB (N = N/A)	Unselected	N/A	Outcomes described as dismal, trial was prematurely terminated due to unexpected lack of efficacy	High toxicity profile with 100% total AEs and >30% serious adverse events Frequent AEs: Marked skin, mucosal, and gastrointestinal toxicities	[208]

**Table 3:** Compounds included in preclinical studies.

Compounds	Molecular Target	Experimental Model	Main Evaluation Parameters	Reported Activity & Experimental Outcomes	Observed Toxicity/Limitations	Ref.
Osimertinib (AZD9291)	Mutant EGFR, EGFRvIII	- In vitro: human GSCs expressing EGFRvIII+: D317, D10-0171, D10-0279, D09-0155, HK301, HK412. - In vivo: heterotopic and orthotopic xenograft mouse models; oral osimertinib (25 mg/kg).	EGFR phosphorylation/signaling, cell viability/proliferation, tumor growth, overall survival, body weight (tolerability)	Osimertinib effectively penetrated the BBB, inhibited EGFRvIII signaling, suppressed tumor growth, and prolonged survival in EGFRvIII-high GB models.	Minimal toxicity (no significant weight loss); toxicity assessment limited; efficacy mainly demonstrated in EGFRvIII-high models.	[111]
Osimertinib (AZD9291)	Mutant EGFR, EGFRvIII	- In vitro: human GB cell lines: U87, U251, U118, LN229, T98G, LN18. - In vivo: orthotopic murine model, intraperitoneally injected or oral administration with 15 mg/kg or 30 mg/kg dose.	EGFR/ERK phosphorylation, cell viability, apoptosis, tumor growth, and overall survival.	Osimertinib overcame primary resistance by continuously suppressing ERK signaling, resulting in reduced tumor growth and prolonged survival in glioblastoma models	Limited toxicity evaluation; findings require clinical validation.	[112]
Tyrphostins (AG1433)	PDGFR	- In vitro: Primary high-grade glioma cultures: 11HGG and 15HGG, exposed to ionizing radiation.	PDGFR expression/phosphorylation, cell viability, clonogenic survival, apoptosis, cell-cycle distribution, radiosensitivity.	PDGFR inhibition enhanced the response to ionizing radiation, decreasing clonogenic survival and increasing apoptosis/radiosensitivity, particularly in PDGFR-expressing cells	No toxicity assessment; limited number of primary HGG cell lines; no in vivo validation.	[140]
Fexagratinib (AZD4547)	FGFR1, FGFR2, and FGFR3	- In vitro: FGFR-dependent human cancer cell lines: KG1a, Sum52-PE, MCF7, and KMS11. - In vivo: Human tumor xenograft mouse models.	FGFR kinase inhibition, receptor phosphorylation, cell proliferation, downstream signaling, antitumor activity, pharmacokinetics/pharmacodynamics.	AZD4547 potently and selectively inhibited FGFR signaling, suppressing tumor cell proliferation and inducing significant antitumor activity in FGFR-driven xenograft models.	Well tolerated in preclinical models; not evaluated in glioblastoma; further clinical validation required.	[151]
Tivantinib (ARQ197)	c-MET, PI3K/Akt/mTOR signaling pathway	- In vitro: U251 and T98MG glioblastoma cells.	Cell viability/proliferation, apoptosis, cell-cycle distribution, migration/invasion, c-MET and PI3K/Akt/mTOR signaling.	Tivantinib inhibited glioblastoma cell proliferation through PI3K/Akt/mTOR pathway suppression; combination with downstream PI3K and mTOR inhibitors enhanced its therapeutic effects.	Limited to in vitro data; no toxicity evaluation; lack of in vivo validation.	[178]
Foretinib	c-MET, VEGFR2, PDGFR, AXL, Tie-2	- In vitro: human glioblastoma cell lines T98G, U87MG and U251.	Cell viability/proliferation, cell-cycle distribution, apoptosis, invasion, c-MET expression/signaling.	Foretinib inhibited glioblastoma cell proliferation and invasion through c-MET inhibition, inducing G2/M cell-cycle arrest and apoptosis.	No toxicity assessment; further in vivo safety evaluation required.	[179]
VGB peptide	VEGFR1, VEGFR2	- In vitro: human umbilical vascular endothelial cells (HUVECs), 4T1 mammary carcinoma tumor cells and U87 glioblastoma cells.	VEGFR1/VEGFR2 inhibition, VEGF-induced endothelial proliferation, migration, tube formation, angiogenesis, tumor growth, metastasis, PI3K/AKT and MAPK/ERK signaling.	Dual blockade of VEGFR1 and VEGFR2 inhibited VEGF-driven angiogenesis, tumor growth, and metastasis by suppressing PI3K/AKT and MAPK/ERK1/2 signaling pathways.	Limited toxicity evaluation; further validation required before clinical translation.	[198]
Voacangine (Voa)	VEGFR2, HIF-1α/ VEGF axis	- In vitro: human umbilical vascular endothelial cells (HUVECs). - In vivo: Chick chorioallantoic membrane (CAM) assay.	Endothelial cell proliferation, migration, tube formation, VEGFR2 phosphorylation, angiogenesis.	Voacangine inhibited VEGFR2 activation and suppressed angiogenesis by reducing endothelial cell proliferation, migration, and tube formation both in vitro and in vivo.	No significant cytotoxicity toward endothelial cells at antiangiogenic concentrations; limited toxicity assessment.	[199]
Voa analogue 19 (V19)	VEGFR2, HIF-1α/ VEGF axis	- In vitro: Glioma U87MG cells and endothelial HUVECs cultures. - In vivo: Nude mice bearing subcutaneous human glioblastoma tumor (U87MG) xenografts were injected intraperitoneally with V19 (10 mg/kg).	VEGFR2 phosphorylation, kinase activity, cell proliferation/cytotoxicity, endothelial cell migration, tube formation, tumor growth.	V19 directly inhibited VEGFR2 kinase activity, suppressed VEGF-induced VEGFR2 phosphorylation and angiogenesis, and induced significant tumor cell death in a mouse xenograft model without cytotoxic effects in endothelial cells.	No cytotoxic effects in HUVECs at active concentrations; limited toxicity evaluation and no long-term safety assessment.	[200]

## 6. Discussion

In addition to intrinsic tumor biology, complex neuro-pharmacokinetic principles dictate the modest clinical efficacy of TKIs in GB. A critical distinction must be made between the total brain drug concentration and the unbound, pharmacologically active fraction. While total tissue measurements often suggest successful delivery, only the unbound drug in the interstitial fluid is capable of crossing tumor cell membranes to inhibit intracellular kinase domains. Active efflux via P-gp and BCRP at the BBB drastically restricts this active fraction, a phenomenon quantified by a low unbound brain-to-plasma concentration ratio. Furthermore, historical reliance on CSF sampling as a surrogate for brain penetration represents a significant translational pitfall. Due to the distinct physiology of the blood–CSF barrier, rapid fluid turnover, and spatial distance from the tumor core, drug concentration in the CSF does not equilibrate with, nor accurately reflect, therapeutic drug penetration into the brain parenchyma or the heterogeneous tumor tissue.

Collectively, these factors demonstrate that, while kinase inhibition persists as a compelling therapeutic strategy, its success in GB depends on overcoming both biological redundancy and drug delivery constraints [33].

### 6.1. Design Strategies to Enhance Brain Delivery of Kinase Inhibitors in Glioblastoma

#### 6.1.1. Physicochemical Optimization for CNS Penetration

**Figure 6:**
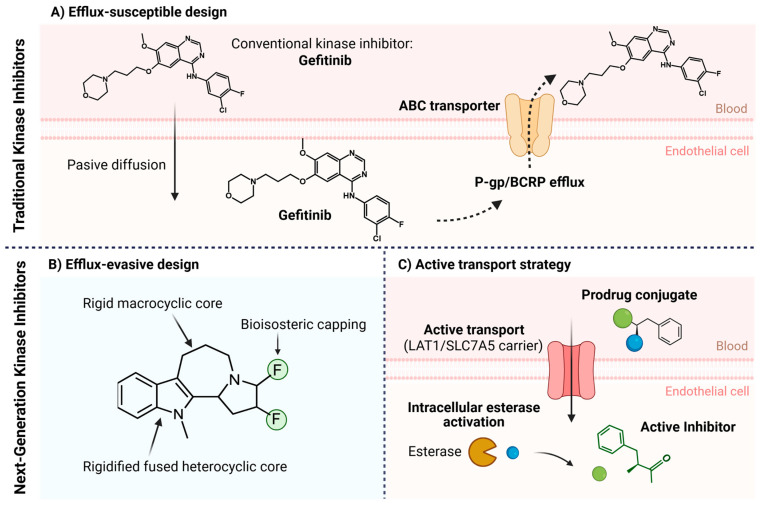
Design strategies to enhance brain delivery of kinase inhibitors. Conventional kinase inhibitors, such as gefitinib, often exhibit limited brain exposure due to active efflux at the blood–brain barrier. Following passive diffusion into endothelial cells, these compounds are recognized by ATP-dependent transporters including P-gp and BCRP, resulting in restricted CNS accumulation (**A**). Next-generation kinase inhibitors employ rational molecular optimization strategies (**B**), including conformational rigidification, reduction in exposed hydrogen-bond donors, and optimization of physicochemical properties such as topological polar surface area (TPSA), thereby minimizing transporter recognition and improving passive permeability. Alternatively, active transport strategies (**C**) employ prodrug conjugate approaches to exploit endogenous carrier systems, such as LAT1, to facilitate BBB translocation and enhance intracellular delivery of kinase inhibitors. Created in BioRender. Hijazi Vega, M. (2026) https://BioRender.com/yfrqft3 (accessed on 16 July 2026).

A central strategy to enhance the therapeutic performance of kinase inhibitors in GB relies on the rational structural optimization of these molecules to facilitate BBB permeation (Figure 6). Although many TKIs exhibit high biochemical potency against their intended targets, their clinical efficacy is frequently limited by insufficient intracranial exposure. This discrepancy has driven the development of medicinal chemistry approaches aimed at aligning physicochemical properties with the stringent constraints imposed by the BBB [209].

From a physicochemical standpoint, passive diffusion across the BBB is primarily governed by molecular weight, lipophilicity, topological polar surface area, and hydrogen-bonding capacity. Compounds with moderate molecular weight, balanced lipophilicity, and low polarity are generally more likely to penetrate the CNS. However, most clinically relevant TKIs are structurally complex and highly functionalized, features that favor target engagement but often compromise brain permeability [209].

Lipophilicity represents a particularly critical parameter within this optimization landscape. While moderate increases in lipophilicity can enhance transcellular diffusion across the BBB, excessive lipophilicity is associated with increased plasma protein binding, peripheral tissue accumulation, and reduced free drug fraction available for CNS distribution. Consequently, successful design does not aim to maximize lipophilicity, but rather to finely tune it within a narrow window that balances permeability with pharmacokinetic stability and safety [209].

In practice, structural optimization is achieved through targeted and often subtle chemical modifications. Reducing the number of non-essential heteroatoms, particularly oxygen and nitrogen atoms, decreases molecular polarity and hydrogen-bonding potential, thereby facilitating passive diffusion. Similarly, the replacement of highly polar functional groups with less hydrophilic bioisosteres allows preservation of target-binding geometry while lowering TPSA. These strategies are frequently combined with modulation of ionization properties, as favoring neutral or weakly ionized species at physiological pH enhances membrane permeability [209].

Another key approach involves the rigidification of molecular structures through reduction in rotatable bonds or cyclisation of flexible chains. Increased rigidity can improve permeability by reducing the entropic penalty associated with membrane partitioning and, in some cases, may also decrease recognition by efflux transporters. However, excessive conformational constraint may impair optimal target binding, underscoring the need for careful structural balance [209].

Fine-tuning of lipophilicity and electronic properties is often achieved through the controlled introduction of hydrophobic substituents, such as alkyl groups or halogens, as well as through modification of aromatic and heterocyclic systems. In parallel, modulation of pKa via electronic effects can shift the ionization state of the molecule, promoting forms that are more conducive to BBB permeation. These adjustments, although subtle, can have a disproportionate impact on overall CNS exposure [210].

Despite these advances, physicochemical optimization remains inherently constrained by competing design requirements. Reducing polarity or hydrogen-bonding capacity may improve brain penetration but can simultaneously weaken target affinity. Similarly, increasing rigidity or lipophilicity may enhance permeability at the expense of solubility, metabolic stability, or safety. Importantly, structural features that favor BBB penetration may still predispose compounds to active efflux, further limiting intracranial accumulation.

Collectively, these considerations highlight that the design of brain-penetrant kinase inhibitors is not a unidimensional optimization problem, but rather a multidimensional balancing act in which potency, permeability, metabolic behavior and efflux liability must be simultaneously reconciled. In GB, where effective drug delivery remains a major barrier to therapeutic success, physicochemical optimization represents a necessary but insufficient strategy, thereby motivating the development of complementary approaches to further enhance CNS exposure.

#### 6.1.2. Minimizing Efflux Transporter Recognition (P-gp and BCRP Evasion)

A major barrier to the clinical efficacy of kinase inhibitors in GB is active efflux at the BBB mediated by ATP-binding cassette transporters, particularly P-glycoprotein and BCRP (Figure 6A). These efflux systems restrict brain accumulation of many anticancer agents by actively exporting structurally diverse xenobiotics from the CNS, thereby severely limiting therapeutic exposure despite adequate systemic potency [211].

From a medicinal chemistry perspective, transporter recognition is strongly influenced by molecular architecture and physicochemical properties. Compounds with high lipophilicity, pronounced conformational flexibility, and extended aromatic surface area are more likely to be recognized as substrates of P-gp and BCRP. In addition, increased hydrogen-bonding capacity and dense heteroatom patterns can enhance interactions with transporter binding pockets, further promoting efflux [210].

Rational design strategies therefore aim to decouple target affinity from efflux susceptibility (Figure 6B). A key approach is the fine-tuning of lipophilicity and molecular rigidity to an intermediate physicochemical space that supports passive diffusion while reducing transporter engagement. In this context, overly hydrophobic and flexible scaffolds are generally disfavored, as they tend to increase P-gp/BCRP substrate recognition, whereas controlled rigidification and balanced polarity can reduce efflux liability without fully compromising BBB permeability [210,211].

Complementary strategies involve the systematic modulation of functional groups involved in transporter recognition. Replacement of highly polar substituents with less hydrogen-bonding bioisosteres and reduction in overall hydrogen-bond donor/acceptor count have been shown to decrease affinity for P-gp and BCRP, provided that the key pharmacophoric interactions with the kinase hinge region are preserved [211].

Importantly, complete avoidance of efflux recognition is often neither achievable nor desirable. Instead, successful CNS-active kinase inhibitors frequently rely on partial evasion of P-gp and BCRP sufficient to permit therapeutically relevant brain exposure. This reflects the broad and promiscuous substrate specificity of these transporters, which limits the feasibility of absolute exclusion strategies [210].

However, optimization in this space is inherently constrained by competing pharmacological requirements. Reducing polarity or increasing rigidity to avoid efflux may adversely affect solubility, oral bioavailability, or target binding affinity. Consequently, effective design requires simultaneous optimization of potency, physicochemical properties, and transporter liability within a narrow therapeutic window [210].

This structural optimization paradigm remains a key determinant in improving brain delivery of kinase inhibitors, although it must be integrated with complementary medicinal chemistry and drug delivery strategies to achieve meaningful therapeutic impact in GB [210].

#### 6.1.3. Prodrug and Chemical Delivery Approaches

Prodrug approaches represent a widely explored strategy to improve the CNS exposure of kinase inhibitors, particularly in GB, where BBB permeability remains a major limiting factor (Figure 6C). This strategy relies on the reversible chemical modification of an active pharmaceutical ingredient to transiently optimize its physicochemical properties, without permanently altering its pharmacologically active core. In cases where direct structural optimization is insufficient to overcome BBB constraints, prodrugs provide an alternative route to enhance brain delivery while preserving target-specific activity upon activation [212,213].

From a chemical perspective, a prodrug is an inactive or less active derivative of a parent compound that undergoes enzymatic or chemical conversion into its active form after reaching the target tissue. In the context of brain tumors, this design principle is exploited to increase BBB permeability by temporarily masking polarity, charge, or hydrogen-bonding capacity, thereby facilitating passive diffusion or transporter-mediated uptake into the CNS. Once within the brain microenvironment, the prodrug is converted into the active kinase inhibitor through endogenous enzymatic processes, ideally resulting in localized drug activation within the tumor site [213].

The common implementation of this strategy involves the incorporation of lipophilic promoieties, which transiently increase the compound’s affinity for lipid environments and enhance membrane permeability. These moieties can improve both oral absorption and brain distribution, provided that their cleavage is efficient and spatially controlled. However, the success of this approach is highly dependent on the predictability of metabolic activation pathways, as incomplete or premature conversion can significantly reduce therapeutic efficacy or shift systemic exposure away from the CNS [212].

Despite their conceptual advantages, prodrug strategies are associated with several intrinsic limitations. These include interindividual variability in enzymatic activity, the risk of premature systemic activation prior to BBB crossing, and the potential formation of metabolites with poorly characterized safety profiles. In addition, prodrug design introduces an additional layer of pharmacokinetic complexity, requiring simultaneous optimization of both the inactive precursor and the active parent compound, which can constrain translational efficiency in drug development [212].

Overall, prodrug strategies constitute a valuable complementary approach to direct structural optimization of kinase inhibitors. Their principal advantage lies in the ability to enhance brain delivery without permanently modifying the active pharmacophore. However, in GB, their clinical applicability is constrained by the need for precise control over stability, activation kinetics, and CNS-selective release, limiting their utility to contexts where conventional medicinal chemistry optimization alone is insufficient to achieve adequate brain exposure [212,213].

### 6.2. Future Research, Prospects and Limitations

#### 6.2.1. Clinical Trials Failure

The clinical translation of small-molecule kinase inhibitors in GB has been historically discouraging, marked by a repetitive pattern of high preclinical expectations followed by systemic failures in human subjects. For decades, the neuro-oncology community routinely attributed the failure of first-generation TKIs, such as erlotinib and gefitinib, almost exclusively to their poor BBB penetration and lack of intracranial accumulation. However, recent clinical trials utilizing next-generation molecules specifically engineered to cross the neurovascular interface have revealed a far more intricate and daunting biological reality. These modern studies demonstrate that achieving adequate intracranial exposure, while structurally necessary, remains modest if the agent is deployed as a single-node monotherapy against an ultra-adaptive oncogenic network.

An example of this paradigm shift is the clinical trajectory of paxalisib (GDC-0084), a dual PI3K/mTOR inhibitor designed specifically for enhanced CNS distribution, exhibiting an outstanding unbound brain-to-plasma ratio. Despite its optimized pharmacokinetics, the evaluation of paxalisib within the international, multi-arm GBM AGILE master protocol (NCT03970447) ultimately revealed that the drug failed to meet its primary endpoint of improving overall survival in newly diagnosed or recurrent GB patients. While the comprehensive design framework of this adaptive trial platform has been thoroughly [214], the top-line negative survival outcomes were disclosed through official registry updates and international clinical symmetry presentations rather than a consolidated primary results publication. The clinical outcomes of paxalisib strongly suggest that targeting the PI3K/Akt/mTOR axis as an isolated monotherapy faces severe biological limitations. Inhibiting this single node often disrupts endogenous negative feedback loops, which may consequently trigger a compensatory hyperactivation of the parallel MAPK/ERK pathway, potentially neutralizing the drug’s therapeutic efficacy.

An identical obstacle has compromised the clinical translation of third-generation EGFR inhibitors, such as osimertinib. While osimertinib has revolutionized the management of EGFR-mutant non-small-cell lung cancer brain metastases due to its exceptional BBB permeability [215], its efficacy in recurrent GB harboring EGFR alterations remains modest and clinically transient. We argue that this therapeutic gap stems directly from the profound spatial and temporal clonal heterogeneity that distinguishes GB from oncogene-addicted epithelial malignancies. As established in a landmark genomic study [216], while EGFR mutations in lung adenocarcinoma represent foundational, truncal alterations present in virtually every malignant cell, EGFR amplifications and the EGFRvIII mutant in GB reside predominantly on highly dynamic extrachromosomal DNA (ecDNA) elements. These ecDNA structures fluctuate rapidly under therapeutic pressure, allowing non-driven or alternative clones within the same tumor mass to outgrow the suppressed population within weeks, presenting a severe hurdle for traditional single-agent approaches.

In parallel with the limitations observed for highly selective agents, the multi-targeted TKI regorafenib achieved a statistically significant, though clinically modest, overall survival benefit compared with lomustine in the phase II REGOMA trial. This clinical response highlights an interesting paradigm in neuro-oncology [207]. Regorafenib targets an exceptionally broad spectrum of kinases, including VEGFR1-3, TIE2, PDGFR, FGFR, and KIT. In our opinion, the relative success of regorafenib is precisely a consequence of its non-selective, promiscuous pharmacological profile. By simultaneously disrupting endothelial angiogenesis through VEGFR inhibition and disabling stromal-to-tumor crosstalk through PDGFR and FGFR blockade, regorafenib achieves what highly specific compounds cannot: the partial collapse of multiple cellular and microenvironmental pillars within the tumor network, preventing immediate adaptation.

To circumvent the historical pattern of clinical failures driven by single-agent therapies and immediate adaptive resistance, we argue that future translational research should increasingly focus on the rigorous preclinical evaluation of rational, co-targeted combinations designed to proactively neutralize compensatory feedback loops. Specifically, rather than deploying single-node blockades that inevitably fail in clinical settings, we propose prioritizing the preclinical co-administration of a dual PI3K/mTOR inhibitor, such as paxalisib, alongside a vertical or parallel pathway blocker, specifically a MEK inhibitor like trametinib. Since blocking the PI3K/Akt/mTOR axis in isolation abruptly dismantles endogenous negative feedback loops and triggers an immediate, compensatory hyperactivation of the parallel MAPK/ERK pathway, this dual-node strategy aims to systematically block the tumor’s primary escape routes. Crucially, given the current stage of development, proposing an immediate clinical trial remains premature. Instead, the primary objective of this preclinical framework should center on identifying and validating robust molecular biomarkers of response to this therapeutic synergy. Characterizing multi-omics signatures that predict which tumor subclones are uniquely vulnerable to this dual inhibition will provide the necessary mechanistic rationale required to stratify patients effectively, thereby replacing empirical clinical expansion with precise, biomarker-driven candidate selection.

#### 6.2.2. Next-Generation Inhibitor Design

A primary limitation within modern medicinal chemistry is that the structural modifications required to maximize a molecule’s affinity for a kinase ATP-binding pocket frequently doom the compound to active efflux extrusion at the BBB interface. To break this historical cycle, we propose that future drug discovery campaigns should complement the traditional pursuit of absolute in vitro nanomolar potency in cell-free assays with a rigorous assessment of cellular contextual biology and multidimensional optimization. Medicinal chemists should treat BBB permeation not as a secondary filter applied late in development, but as the foundational structural framework upon which target affinity is subsequently built.

To achieve meaningful intracranial exposure, the design of novel kinase inhibitors should implement enforced conformational constraint through macrocyclization or the synthesis of rigidified fused heterocyclic cores (Figure 6B). Linear, flexible small molecules possess a large number of rotatable bonds, which imposes a heavy entropic penalty during lipid bilayer translocation and allows the molecule to dynamically adapt its shape to fit into the binding pockets of efflux transporters. Restricting molecular flexibility through structural cyclization locks the scaffold into its bioactive conformation, minimizing the entropic barrier to passive diffusion while simultaneously altering the precise spatial geometry recognized by P-glycoprotein and BCRP. This partial evasion of active efflux ensures that a higher fraction of the administered drug remains within the brain parenchyma [217].

Furthermore, next-generation chemical design should ideally prioritize the systematic bioisosteric capping of exposed polar handles to tightly control the molecule’s overall electrostatic properties. The presence of exposed hydrogen-bond donors, such as free hydroxyl (OH) or amine (NH) groups, forces the molecule to form a dense hydration shell in plasma, requiring a high energetic cost to desolvate before it can cross the endothelial cell membrane. Medicinal chemists often consider replacing these problematic groups with fluorine atoms, trifluoromethyl groups (CF3), or compact, less polar heterocycles such as oxetanes. This strategy can help maintain the topological polar surface area well below the generally accepted neuro-pharmacological threshold of 90 Å^2^ without necessarily sacrificing the key hydrogen-bonding interactions required to engage the kinase hinge region. Minimizing the TPSA while optimizing the ionization constant (pKa) ensures that the compound exists predominantly as an uncharged, lipophilic species at physiological pH, drastically increasing transcellular passive diffusion.

Importantly, these structural adjustments must be evaluated within a multi-parameter optimization framework, as optimizing a scaffold solely for BBB permeation often introduces severe pharmacokinetic trade-offs. For instance, design strategies aimed at reducing hydrogen bond donors or excessively increasing lipophilicity to favor passive endothelial diffusion can inadvertently impair solubility, accelerate hepatic metabolic clearance, decrease gastrointestinal oral absorption, or introduce unpredictable off-target systemic toxicities. Therefore, balancing CNS exposure with a sustainable safety and efficacy profile remains an essential requirement for successful clinical translation.

Finally, when the structural activity relationship of a highly potent kinase inhibitor is rigid and cannot be modified without a substantial loss of target engagement, the utilization of sophisticated prodrug strategies represents a highly viable alternative. Rather than attempting direct modifications of the active pharmacophore, researchers can temporarily mask problematic polar groups by attaching cleavable lipophilic promoieties or targeting endogenous transporter systems. Exploiting solute carriers highly expressed at the BBB endothelium, such as the Large Neutral Amino Acid Transporter 1 (LAT1, encoded by SLC7A5), allows highly functionalized structures to cross the neurovascular interface as a neutral “trojan horse” [218] (Figure 6C). Once inside the brain parenchyma or the tumor interstitial space, endogenous esterases or intracellular enzymes cleave the promoiety, releasing the active multi-kinase inhibitor directly at the disease site. In our view, while prodrug designs add significant pharmacokinetic complexity, they represent an alternative path to rescue exceptional chemical scaffolds that are otherwise completely excluded by the BBB.

From a cutting-edge medicinal chemistry perspective, we maintain that the design of novel candidate inhibitors may be governed by an uncompromising, multidimensional structural roadmap. We propose that future chemical scaffolds should adopt macrocyclized architectures or rigidified fused indazole or pyrrolopyrimidine heterocyclic cores. These are specifically engineered to lock the bioactive conformation and suppress the geometric flexibility recognized by P-gp and BCRP efflux pumps. We propose establishing a SAR operational threshold where the TPSA is tightly maintained between 70 and 85 Å^2^, logD is firmly tuned between 2.0 and 2.5, and a radical bioisosteric capping of exposed hydrogen-bond donor protons (such as free -OH or -NH groups) is performed using fluorine atoms, trifluoromethyl groups (CF_3_), or compact oxetane rings. For highly potent nanomolar in vitro scaffolds whose core molecular connectivity cannot be altered without a total loss of target engagement, we propose the strategic development of prodrugs conjugated with branched amino acids (such as L-phenylalanine). This strategy temporarily masks the drug to potentially exploit active transport across the neurovascular endothelium via the LAT1 (*SLC7A5*) transporter, ensuring a “trojan horse” effect that delivers a massive release of the active inhibitor within the brain parenchyma upon cleavage by endogenous intracellular esterases.

#### 6.2.3. Promising Combinatorial Strategies

Growing evidence suggests that GB is unlikely to be successfully managed by a single pharmacotherapy alone, regardless of the compound’s capacity to penetrate the BBB. Because this malignancy thrives on signaling redundancy and cellular plasticity, the future of targeted therapy belongs predominantly to rational combination regimens designed to hit both the primary driving oncogenic kinases and the downstream adaptive mechanisms responsible for therapy resistance. We identify three distinct combinatorial strategies as the most scientifically promising lines of research to transition the field from single-node inhibition to true network oncology.

The first highly promising line involves combining brain-penetrant KIs with DNA Damage Repair (DDR) inhibitors to exploit the biological concept of synthetic lethality. When GB cells are challenged with kinase inhibitors, they undergo severe oncogenic stress and frequently upregulate parallel survival pathways, including the homologous recombination and non-homologous end-joining DNA repair machinery, to survive concurrent chemotherapy or radiation. Combining multi-targeted TKIs with highly brain-penetrant poly(ADP-ribose) polymerase (PARP) inhibitors, such as pamiparib or niraparib, or selective ATR/CHK1 inhibitors, creates an intolerable level of replication stress. This strategy forces highly resilient GSCs into catastrophic mitotic failure during standard temozolomide chemotherapy, successfully bypassing their intrinsic radio- and chemoresistance mechanisms [219].

The second critical line of research requires the synchronization of next-generation KIs with immune checkpoint modulation to dismantle the tumor’s protective cellular shield. GB is archetypally a cold tumor, characterized by an immunosuppressive microenvironment that is heavily dominated by M2-polarized tumor-associated macrophages (TAMs) and myeloid-derived suppressor cells. This immunosuppressive state is actively sustained by autonomous signaling networks within the tumor niche, driven primarily by colony-stimulating factor 1 receptor (CSF1R), STAT3, and the aryl hydrocarbon receptor (AHR) axes. As demonstrated in the study published by Pyonteck et al. [220], targeting these microenvironmental kinases can successfully re-educate TAMs toward a pro-inflammatory phenotype. We hypothesize that combining a highly brain-penetrant KI capable of blocking the CSF1R or JAK/STAT cascade with anti-PD-1 or anti-CTLA-4 immunotherapies could potentially convert a cold, immunologically silent GB into a hot, T-cell-susceptible tumor, enabling robust systemic immune clearance within the CNS parenchyma.

The third line of research addresses the physical limitations of drug delivery by pairing optimized multi-kinase inhibitors with advanced mechanical disruption techniques. When a multi-targeted chemical scaffold is too large or complex to be optimized for autonomous BBB penetration, combining it with physical delivery systems such as Convection-Enhanced Delivery (CED) or Magnetic Resonance-guided Focused Ultrasound (MRgFU) offers an unparalleled therapeutic advantage. As validated in clinical settings, the application of low-frequency MRgFU in combination with systemic microbubbles transiently, safely, and reversibly disrupts the endothelial tight junctions within the target volume [221]. While MRgFU transiently disrupts endothelial tight junctions to permit the extravasation of larger or highly polar molecular entities, it does not render their physicochemical properties irrelevant. Crucially, parameters such as molecular weight, charge, and TPSA still strictly dictate the drug’s subsequent interstitial diffusion within the brain parenchyma, its dynamic retention within the heterogeneous tumor mass, and its overall rate of tissue clearance.

**Figure 7:**
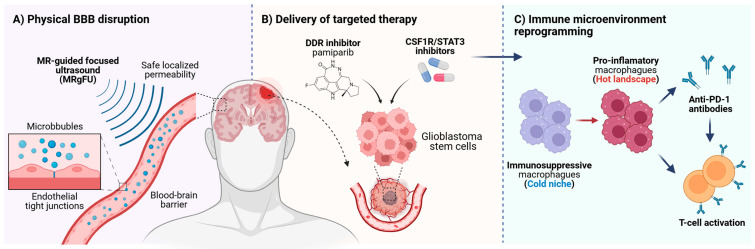
Overview of an integrated multimodal strategy to overcome glioblastoma network plasticity. Transient disruption of the blood–brain barrier using MR-guided focused ultrasound (MRgFUS) and circulating microbubbles enhances localized permeability and facilitates therapeutic delivery to the tumor site (**A**). Increased drug penetration enables the administration of targeted therapies directed against GSCs, including DNA damage response inhibitors such as pamiparib and inhibitors of pro-survival signaling pathways, including CSF1R/STAT3-targeted agents, thereby potentially promoting tumor cell vulnerability and therapeutic response (**B**). Concurrently, modulation of the tumor immune microenvironment promotes the transition from immunosuppressive macrophages to pro-inflammatory phenotypes, enhancing T-cell-mediated antitumor responses and improving the efficacy of anti-PD-1 immune checkpoint blockade (**C**). Created in BioRender. Hijazi Vega, M. (2026) https://BioRender.com/bot7ez6 (accessed on 16 July 2026).

To translate these theoretical concepts into robust preclinical or clinical synergy, we propose a three-pronged framework designed to guide future clinical protocol design (Figure 7). This regimen proposes fusing the temporary physical disruption of the BBB via low-frequency MRgFU and systemic microbubbles (Figure 7A). Immediately following the safe, mechanical opening of endothelial tight junctions within the infiltrative margins, we propose infusing a highly synergistic molecular combination consisting of a brain-penetrant DNA damage repair (DDR) inhibitor, such as pamiparib, to exploit synthetic lethality, alongside a multi-kinase inhibitor explicitly targeting the stromal CSF1R/STAT3 signaling axes (Figure 7B). This strategy is designed not only to crush the radioresistance mechanisms of GSCs by forcing catastrophic mitotic failure, but also to actively re-educate immunosuppressive TAMs toward a pro-inflammatory M1 phenotype (Figure 7C). This repolarization paves the way for anti-PD-1 or anti-CTLA-4 monoclonal antibodies, administered in the same clinical window, to execute a robust and systemic immune clearance within the CNS parenchyma. It must be emphasized that the strategic integration discussed herein represents a speculative model and a hypothetical proposal rather than an established clinical therapy. While the mechanistic rationale is supported by preliminary cross-pathway crosstalk data, this conceptual framework requires extensive validation in preclinical models before any translational implementation can be considered.

#### 6.2.4. Limitations

To bridge the translational gap between preclinical success and clinical failure, the scientific community must confront and resolve major methodological errors currently embedded within neuro-oncology research. For decades, preclinical drug discovery has relied heavily on commercial, serum-cultured in vitro GB cell lines, most notably U87MG and T98G. As rigorously demonstrated by Ledur et al. [222], prolonged cultivation in high-serum environments forces these cells to undergo severe genomic drifting, irreversible transcriptomic alterations, and a complete loss of the original patient tumor’s signaling architecture. We suggest that traditional serum-dependent models can sometimes offer a limited representation of kinase sensitivity, indicating that they should ideally be integrated with or complemented by patient-derived GSC spheres to ensure greater translational relevance.

Furthermore, standard subcutaneous or classical cell-line-derived orthotopic xenograft (CDX) murine models should be complemented with other preclinical models to predict clinical efficacy. Subcutaneous models completely lack a BBB and are exposed to a foreign peripheral stromal environment, while classical intracranial CDX models do not completely replicate the complex human tumor-stromal crosstalk and the highly specialized human neurovascular unit. We argue that while conventional cell lines remain highly valuable tools due to their excellent reproducibility and utility in high-throughput screenings, preclinical evaluation of any candidate kinase inhibitor may be complemented with patient-derived xenograft (PDX) platforms. Notably, these pharmacodynamic studies should be coupled with advanced analytical tools such as Matrix-Assisted Laser Desorption/Ionization Mass Spectrometry Imaging (MALDI-MSI) that offer high-resolution spatial mapping of drug distribution across heterogeneous tumor zones, allowing researchers to visualize whether a compound reaches the necrotic core or the infiltrative margin [55]. However, it must be clarified that MALDI-MSI measures the total local drug concentration within a given tissue voxel and cannot inherently distinguish between the bound and unbound fractions.

## 7. Methods

A comprehensive literature search was conducted across the PubMed, Scopus, and Google Scholar databases to identify relevant peer-reviewed articles published up to 2026, prioritizing updated bibliography. The search matrix utilized combinations of the following keywords: “glioblastoma”, “kinase inhibitors”, “blood–brain barrier”, “efflux transporters”, “P-glycoprotein”, “BCRP”, “medicinal chemistry optimization”, and “combinatorial therapy”. Only articles published in the English language were included. Priority was given to high-impact studies, clinical trial reports, and foundational neuro-pharmacokinetics literature, ensuring an objective, multi-disciplinary synthesis of the current translational landscape.

## 8. Conclusions

The transition of kinase inhibitors from highly promising preclinical candidates to successful clinical therapies in GB represents one of the most complex challenges in modern oncology. Reflecting on the molecular, structural, and pharmacological evidence analyzed throughout this review, we present our expert commentary on the core realities of the field and the strategic steps required to address current therapeutic challenges.

The network paradigm offers a more robust framework than single-target inhibition: GB possesses an inherently redundant, fluid signaling architecture. Targeting a single oncogenic driver, such as wild-type EGFR or the EGFRvIII mutant, is fundamentally insufficient because the tumor dynamically remodels its kinome, immediately rerouting survival signals through parallel RTKs (like MET or PDGFR). Future clinical success belongs predominantly to multi-kinase orchestration strategies capable of shutting down these alternative bypass routes simultaneously before resistance clones expand.

Decoupling affinity from efflux liability is critical: Medicinal chemistry is currently trapped in a structural paradox. The exact features optimized to maximize intracellular hinge-binding affinity (namely rigid heterocyclic planarity, core aromaticity, and basic nitrogen profiles) are the precise molecular fingerprints recognized by the P-gp and BCRP efflux pumps at the BBB. To achieve therapeutic intracranial drug concentrations, drug design should ideally transition toward partial evasion strategies, prioritizing conformational rigidification and bioisosteric modifications that favor passive transcellular diffusion even at the expense of nominal in vitro potency.

Strategic allocation of prodrug architectures: While chemical delivery systems and prodrugs offer an elegant mechanism to transiently mask problematic polar groups and cross the neurovascular interface, they introduce severe pharmacokinetic and regulatory hurdles, including interindividual enzymatic variability and peripheral toxicity risks. Consequently, prodrug development should not be used as a default design choice, but rather as a rescue strategy reserved exclusively for highly potent scaffolds whose structural plasticity has been entirely exhausted.

Eradicating the tumor reservoir via cellular and environmental co-targeting: Eradicating the rapid proliferation of the bulk tumor mass will never prevent relapse if the quiescent reservoir remains untouched. Kinase inhibitor regimens should be strategically synchronized with conventional genotoxic therapies to target the self-renewal networks of glioma stem cells (Notch, Wnt, Hedgehog) and the microenvironmental survival cascades (NF-κB, JAK/STAT) triggered by hypoxia and immunosuppressive macrophage cross-talk.

Ultimately, we conclude that overcoming the clinical inefficacy of targeted therapies in GB requires a fundamental shift in perspective. Current evidence suggests that kinases should no longer be viewed as isolated targets and should begin to be treated as interconnected nodes within a living, highly adaptive network. Integrating CNS-penetrant chemical properties with multi-lineage combination oncology could potentially enrich the therapeutic landscape of this devastating disease.

## Data Availability

The original contributions presented in this study are included in the article. Further inquiries can be directed to the corresponding authors.

## Abbreviations

The following abbreviations are used in this review:

5-ALA	5-Aminolevulinic acid
ABC	ATP-binding cassette
BBB	Blood–brain barrier
BCRP	Breast cancer resistance protein
BTB	Blood–tumor barrier
CNS	Central nervous system
CSF	Cerebrospinal fluid
DDR	DNA damage response
EGFR	Epidermal growth factor receptor
FGFR	Fibroblast growth factor receptors
FGS	Fluorescence-guided surgery
GB	Glioblastoma
GDNF	Glia-derived neurotrophic factor
GSCs	Glioma stem cells
IDH	Isocitrate dehydrogenase
IMRI	Intraoperative magnetic resonance imaging
mAbs	Monoclonal antibodies
MET	Mesenchymal–epithelial transition factor receptor
MRPs	Multidrug resistance-associated proteins
MW	Molecular weight
NGS	Next-generation sequencing
NSCs	Neural stem cells
NSCLC	Non-small-cell lung cancer
NVU	Neurovascular unit
OS	Overall survival
P-gp	P-glycoprotein
PDGFR	Platelet-derived growth factor receptors
PFS	Progression-free survival
ROS	Reactive oxygen species
RT	Radiotherapy
RTKs	Receptor tyrosine kinases
SAR	Structure–activity relationship
smKIs	Small-molecule protein kinase inhibitors
TAMs	Tumor-associated macrophages
TCGA	The Cancer Genome Atlas
TKIs	Tyrosine kinase inhibitors
TME	Tumor microenvironment
TMZ	Temozolomide
TPSA	Topological polar surface area
VEGFRs	Vascular endothelial growth factor receptors
WES	Whole-exome sequencing
WHO	World Health Organization
